# Novel G9 rotavirus strains co-circulate in children and pigs, Taiwan

**DOI:** 10.1038/srep40731

**Published:** 2017-01-18

**Authors:** Fang-Tzy Wu, Krisztián Bányai, Baoming Jiang, Luke Tzu-Chi Liu, Szilvia Marton, Yhu-Chering Huang, Li-Min Huang, Ming-Hui Liao, Chao A. Hsiung

**Affiliations:** 1 Center for Diagnostics and Vaccine Development, Centers for Disease Control, Taipei, Taiwan; 2 Institute for Veterinary Medical Research, Centre for Agricultural Research, Hungarian Academy of Sciences, Budapest, Hungary; 3 Centers for Disease Control and Prevention, Atlanta, Georgia, USA; 4 Department of Pediatrics, Chang Gung Children’s Hospital, Chang Gung Memorial Hospital, Chang Gung University, College of Medicine, Taoyuan, Taiwan; 5 Department of Pediatrics, National Taiwan University Hospital, College of Medicine, National Taiwan University, Taipei, Taiwan; 6 College of Veterinary Medicine, National Pingtung University of Science Technology, Taiwan; 7 Institute of Population Health Sciences, National Health Research Institutes, Zhunan, Taiwan

## Abstract

Molecular epidemiologic studies collecting information of the spatiotemporal distribution of rotavirus VP7 (G) and VP4 (P) genotypes have shown evidence for the increasing global importance of genotype G9 rotaviruses in humans and pigs. Sequence comparison of the VP7 gene of G9 strains identified different lineages to prevail in the respective host species although some of these lineages appear to be shared among heterologous hosts providing evidence of interspecies transmission events. The majority of these events indicates the pig-to-human spillover, although a reverse route of transmission cannot be excluded either. In this study, new variants of G9 rotaviruses were identified in two children with diarrhea and numerous pigs in Taiwan. Whole genome sequence and phylogenetic analyses of selected strains showed close genetic relationship among porcine and human strains suggesting zoonotic origin of Taiwanese human G9 strains detected in 2014–2015. Although the identified human G9P[19] and G9P[13] rotaviruses represented minority strains, the repeated detection of porcine-like rotavirus strains in Taiwanese children over time justifies the continuation of synchronized strain surveillance in humans and domestic animals.

Rotavirus A (RVA) is the major cause of acute gastroenteritis in infants and young children worldwide. Classified into the family *Reoviridae*, RVA strains have an 11-segmented double-stranded RNA genome with a coding capacity of 6 structural proteins (VP1 to VP4, VP6, and VP7) and 6 nonstructural proteins (NSP1 to NSP6)^1^. Classification below the species level includes the assignment of G and P types based on differences of the outer capsid proteins VP7 and VP4, respectively, the primary antigens that induce neutralizing antibody in natural infection or vaccination. Recent extension of the original genotyping system to assign the whole genome based genotype constellation, where Gx-Px-Ix-Rx-Cx-Mx-Ax-Nx-Tx-Ex-Hx designates the genotypes of the VP7-VP4-VP6-VP1-VP2-VP3-NSP1-NSP2-NSP3-NSP4-NSP5/6 protein coding genes, was introduced to better understand the relationship among homologous and heterologous rotavirus strains^2^. Extensive whole genome sequencing studies uncovered that typical human RVA strains carry the G1P[8], G3P[8], G4P[8] or G9P[8] neutralization antigen combinations on a genotype 1 backbone gene constellation (i.e. I1-R1-C1-M1-A1-N1-T1-E1-H1) or the G2P[4] combination on a genotype 2 constellation (i.e. I2-R2-C2-M2-A2-N2-T2-E2-H2)^3^. In other host species the epidemiologically relevant G-P combinations may differ significantly, although some overlaps in the backbone gene constellations can be seen. For example, in pigs frequently identified genotypes, such as G5P[7], G4P[6] or G9P[23], are typically expressed on an I5/I12-R1-C1-M1-A8-N1-T1/T7-E1-H1 genotype constellation^4^,^5^.

In 2006 the Department of Health (current Ministry of Health and Welfare) in Taiwan licensed the pentavalent RotaTeq and the monovalent Rotarix vaccines^6^ and intensified rotavirus strain surveillance. In addition to the common human rotavirus genotypes, the Taiwanese rotavirus strain surveillance network has detected several unusual RVA strains (e.g. genotype P[6] and P[19] RVA strains related to porcine RVA strains) in the pre vaccine era^7^,^8^. These findings were somewhat unexpected and prompted epidemiological pilot studies on animal rotaviruses to be launched when human RVA strain surveillance was reinitiated during 2014–2015.

Although human G9 RVA strains have been isolated sporadically since early 1980s, it went unnoticed until the mid-1990s, when it reemerged to become one of the globally important human RVA VP7 genotype besides G1, G2, G3, G4, and G12^9^,^10^. According to WHO Global RVA Surveillance Network, as of 2013, G9 strains, most often found in combination with P[8] and, less commonly, P[4] and P[6] VP4 genotypes, account for approximately 7.4% of RVA infections globally. While G9 RVAs are prevalent in South Asia and Middle East (20.3%), they are less common in Southeast Asia (5.9%)^11^. The majority of circulating human G9 strains is thought to have descended from a single sublineage around late 1980s following a putative interspecies transmission event between pigs and human in mid-1980s^12^. This newly emerging sublineage has been found to differ from other lineages that include the early human G9P[8] strains (such as WI61, F45, and G2275) and almost all porcine G9 strains, including the porcine-origin human G9P[19] strains (such as Mc323 and RMC321)^12^,^13^. In pigs, genotype G5 (and G5P[7] in particular) is the most common antigen type worldwide (with a relative detection rate from 41.7% to 77.2%); in Asia, G9 RVAs strains represent the second most common genotype in pigs with a detection rate of 11.4%, while in the Americas G9 strains are less commonly detected (2.3%)^14^. A study conducted in Japan from 2001–2002 concluded that G9 strains were predominant among diarrheic piglets in Japanese pig farms and demonstrated the close genetic relationship between these porcine strains and some Japanese human G9 strains isolated in 1999–2000^12^,^15^. In selected studies from various countries (including Korea, Thailand, Belgium, Italy, USA, and Brazil) the predominance of G9 strains in pigs has also been reported^4^,^13^,^16^,^17^,^18^,^19^. Porcine G9 strains usually are found in combination with P[7], P[13], P[19], and P[23]. With the exception of genotype G9P[19], these antigen combinations have not been detected in humans^14^,^20^. Nonetheless, analysis of full genomic sequences determined for unusual porcine-like human G9 strains has raised the possibility of interspecies transmission events between the two host species by demonstrating shared genetic lineages of selected rotavirus genes^21^,^22^,^23^.

In the present study, we analyze the genotype constellation of selected human G9P[19] RVA strains and describe a human G9P[13] RVA strain that was hitherto unknown in human infection. Our analysis provides strong evidence that majority of gene segments, including the backbone genes, in the newly identified human G9 strains can be deduced from porcine RVA strains that co-circulated with the human G9 RVAs in parts of Taiwan. In addition, we demonstrate that many of these genes cluster in genetic lineages that are distinct from those described in other parts of the world, indicating a complex history of reassortment and spillover events between the two hosts in Taiwan.

## Results and Discussion

### Genotype G9 rotaviruses and enteric co-infections in humans and pigs

The Taiwanese rotavirus strain surveillance network was initiated in 2004 and children aged under 5 years admitted to hospital with acute gastroenteritis (AGE) were enrolled from 3 sentinel hospitals. From 2004 to 2011, a total of 9,566 stool specimens were collected and 2,038 (21.3%) tested positive for rotavirus by EIA and VP4/VP7 RT-PCR. Since 2014, the surveillance has been expanded to 10 sentinel sites^24^,^25^. In 2014–2015, a total of 2,444 AGE cases were enrolled out of which 222 (9.1%) were found positive for RVA. Between 2005 and 2011, 250 (12.3%) specimens were identified as G9 by PCR-sequencing, including 2 G9P[19] and 1 G9P[6] strains. One of the 2 G9P[19] sample was selected for viral full-length gene analysis. In 2014–2015, among 222 rotavirus positive samples 32 (14.4%) were genotyped as G9 including 1 G9P[19] and 1 G9P[13] strain. Both G9 strains from 2014–2015 were selected for genetic analysis. The primary aim was to determine the full or near-complete genomes for the selected unusual human G9 strains; in practice, for one human strain, D152, only partial sequence could be determined for VP3 (1–761 bp) and VP4 (33–1615 bp) genes because only limited amount of specimens was available.

Furthermore, 1,350 and 1,080 stool samples were collected from 17 pig farms across Taiwan in 2014 and 2015, respectively, as part of epidemiologic surveillance for animal enteropathogens. In total, 104 samples (4.2%) were positive for rotavirus by a combination of EIA and RT-PCR. Among these, 29 (18 in 2014 and 11 in 2015) were found to contain G9P[13], G9P[19], or G9P[23] RVA strains. Almost all specimens were collected from healthy pigs of various ages.

In the relatively large number of G9 positive cases no other enteropathogenic viruses were detected and only three samples were found to be positive for pathogenic enteric bacteria. Two porcine samples, 3–17 and 3–18, tested positive for Enterotoxigenic E. coli (ETEC) whereas one human sample, containing the strain D152, was positive for Salmonella spp.

### Phylogenetic analysis of novel Taiwanese G9 strains

**Figure 1:**
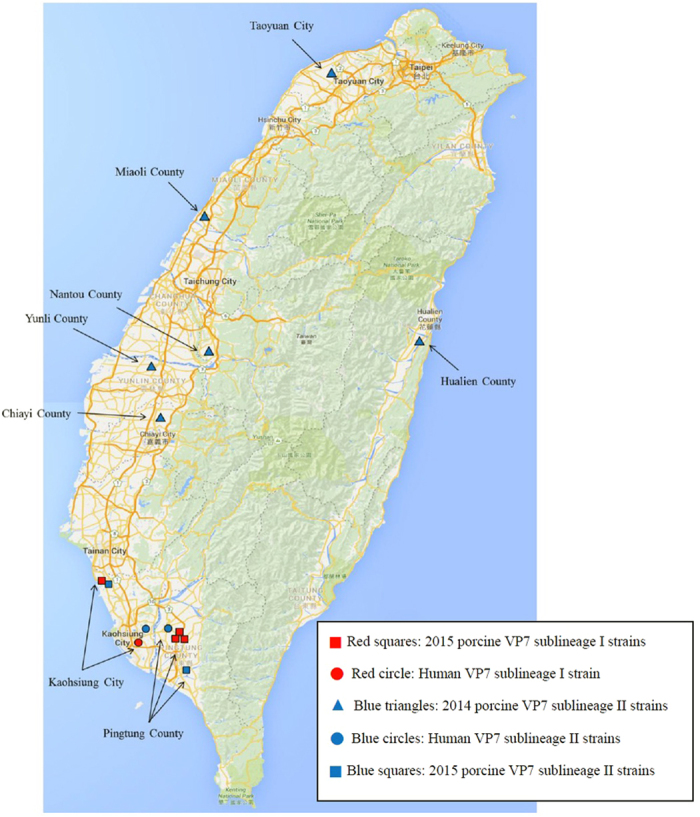
Map of locations in which G9 positive pig farms and residences of patients are located. Map modified from Google Maps: Map data ©2015 Google, ZENRIN, with Adobe Photoshop CS 8.0

**Figure 2:**
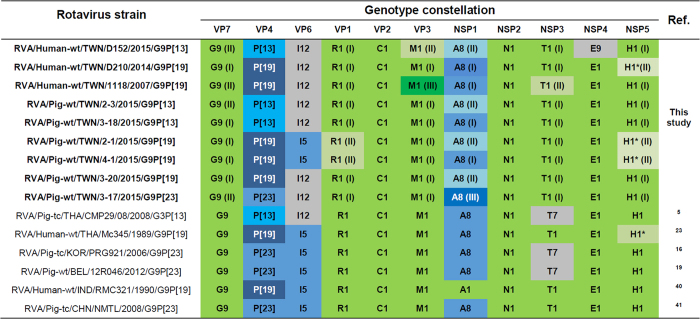
Genotype constellations of Taiwanese porcine and human G9 RVA strains. Human and porcine strains with similar genotype constellations are included for comparison. Wa-like genotypes are indicated in shades of green, typical porcine genotypes are indicated in shades of blue, and grey represent genotypes that are unusual in human or porcine strains. Strains characterized in this study are indicated in boldface. Roman numerals in parentheses after genotype in strains from this study represent the cluster as identified in phylogenetic trees in Figs 4 and 5. Asterisks indicate rearranged NSP5^40^,^41^.

Of interest, the synchronized rotavirus strain surveillance study conducted in humans and pigs detected shared rotavirus genotypes in nearby districts/townships in Southern Taiwan in 2014 to 2015 (Fig. 1 and Supplemental Fig. S1). This finding provided a unique opportunity to compare co-circulating human and porcine RVA strains sharing antigen combinations (additional details are available in Table 1, Fig. 2 and Supplemental Table S2).

**Figure 3:**
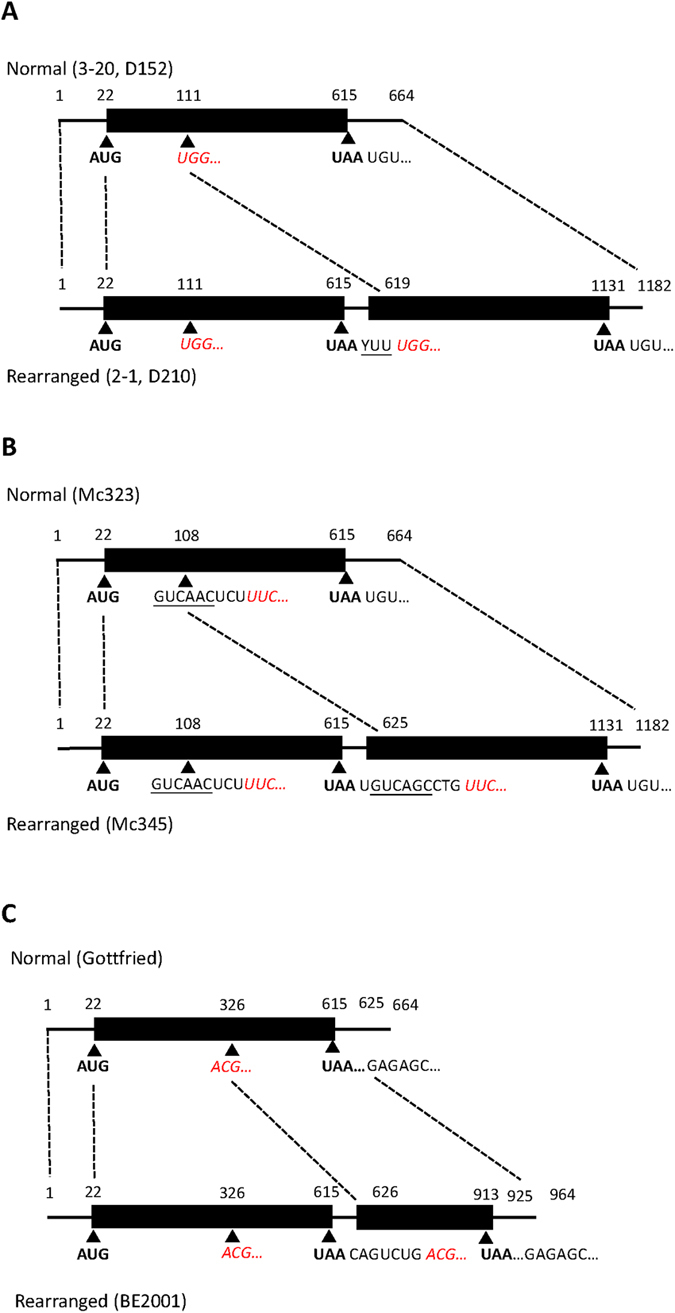
Structure of normal and rearranged NSP5 in (**A)**. Taiwanese porcine strains 3-20, 2-1 and human strains D152, D210 compared to (**B)**. Thai human strains Mc 323 and Mc345, and (**C)** porcine strains Gottfried and BE2001. Start and stop codons are in bold, YUU and direct repeat motifs are underlined, and beginning of duplicated sequence in red italics. Accession numbers for NSP5 sequences used: 3-20: KU739956, 2-1: KU739916, D152: KU740018, D120: KU740026, Mc323: JN872347, Mc345: JN872348, Gottfried: GU199491, BE2001: JQ993328.

An overview of the identified genotype constellations in Fig. 2 shows a conservative combination of backbone genes (i.e. I5/I12-R1-C1-M1-A8-N1-T1-E1/E9-H1) with some variation in the VP6, NSP4 and NSP5 genes. In general, while the VP6 and NSP4 genes of identified G9 RVA strains belonged to different genotypes (i.e. I5/I12 and E1/E9), the primary differences in the NSP5 gene were seen in the length and structure of the respective gene segments (Fig. 3).

**Figure 4:**
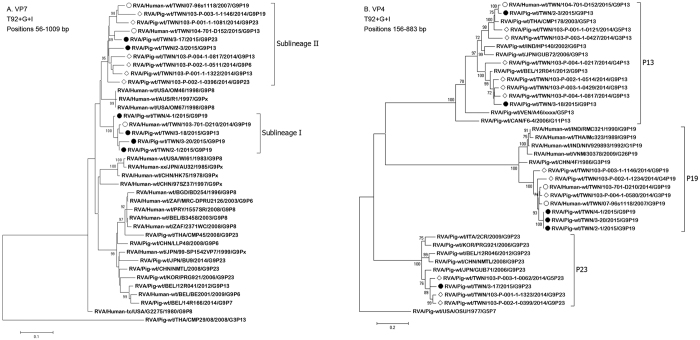
Maximum likelihood phylogenetic tree the VP7 gene (**A**) and the VP4 gene (**B**) of Taiwanese G9 strains identified in humans and pigs. The G9 VP7 tree was reconstructed using partial gene sequences (VP7, nt 56–1009; VP4, nt 156–883). In both reconstructions the Tamura 3-parameter substitution model with gamma and invariant sites was used with 1,000 bootstrap replicates. Only >75% bootstrap values are shown. Closed circles indicate Taiwanese porcine strains from 2015; open circles indicate Taiwanese human strains; open diamonds denote Taiwanese porcine strains from 2014 used here as reference.

Phylogenetic analysis of the G9 VP7 gene revealed that the contemporary Taiwanese human G9 strains fell into two distinct genetic lineages most closely related to American human G9 strains from 1998 (Fig. 4A). One Taiwanese lineage was represented by a single historic G9P[19] strain detected in Taiwan during 2007^8^, while the other lineage had no known representative strains in literature. Both novel G9 VP7 lineages were found among the selected Taiwanese porcine and human G9 strains suggesting that these unique Taiwanese G9 VP7 lineages may be spreading in local pig farms. Phylogenetic analysis of the VP4 gene indicated that the novel human G9P[13] strain shared the VP4 gene with contemporary Taiwanese porcine strains which showed relatedness with a Thai porcine P[13] strain. The other porcine P[13] strain was more closely related to a Belgian porcine P[13] strain (Fig. 4B). All Taiwanese human and porcine P[19] and P[23] strains fell into a common major cluster which differed from porcine and human P[19]/P[23] strains identified in other Asian countries, although geographically, the closest relative in each case was to another East Asian porcine RVA, the G9P[23] strain GUB71 from Japan and the G9P[19] strain 4 F from China (Fig. 4B)^8^.

**Figure 5:**
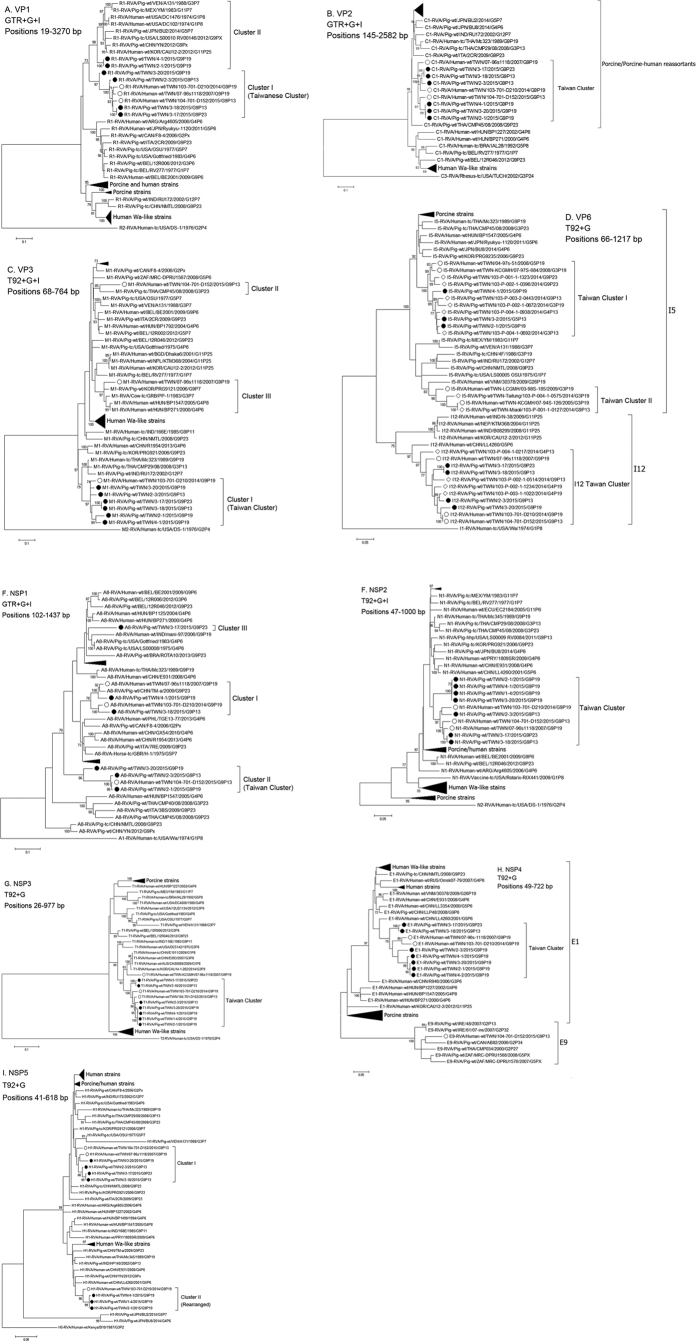
Condensed phylogenetic trees of (**A**) VP1, (**B**) VP2, (**C**) VP3, (**D**) VP6, (**E**) NSP1, (**F**) NSP2, (**G**) NSP3, (**H**) NSP4, and (I) NSP5 were constructed with MEGA6 using neighbor-joining (maximum composite likelihood) or maximum likelihood method with 1,000 bootstrap replicates using the best-fit substitution models determined by the built-in model test application: GTR = General Time Reversible, T92 = Tamura 92, HKY = Hasegawa-Kishino-Yano 85, TN93 = Tamura-Nei 93, G = gamme sites, I = invariant sites. The region within each of the gene segment used for each analysis is also shown. Open circles indicate Taiwanese human strains, closed circles denote Taiwanese porcine strains from 2015, open diamonds denote Taiwanese porcine strains from 2014.

Phylogenetic analysis of the VP1 gene showed close relatedness of the Taiwanese strains to two human G1P[8] strains from 1970 s, a recent unusual Korean G11P[25] strain, and some porcine strains from different time periods (Fig. 5A, Supplemental Fig. S2A). Taiwanese strains could be divided into two clusters: one cluster (cluster I) contains exclusively Taiwanese human and porcine strains while the other (cluster II) contains the two porcine strains from Southern Taiwan. These clusters are distant from typical Wa-like human strains, suggesting a porcine origin in all Taiwanese VP1 genes sampled here. The VP2 genes clustered into a single common lineage that shared a maximum of 92% nucleotide identity with the closest strain with available VP2 sequence in GenBank (Fig. 5B, Supplemental Fig. S2B, Supplemental Table S2). The VP2 gene of the Taiwanese RVAs are more closely related to porcine/porcine-like human strains than to Wa-like human strains. In contrast to VP1 and VP2 genes, VP3 segments are genetically more heterogenous (Fig. 5C, Supplemental Fig. S2C). As with the VP1 and VP2, all porcine strains and a single human G9P[19] strain, D210, are grouped within a distinct cluster (cluster I) that is related to other Asian porcine and porcine-like human strains. The two other human strains are each located in different branches within the tree; the G9P[13], D152, clustered with a Thai G3P[23] porcine strain (cluster II), while a G9P[19] strain,1118, isolated in 2007 was related to Korean porcine and Hungarian human strains (cluster III). Concerning the VP6 gene, 2 I genotypes, I5 and I12, which are relatively closely related to each other, were observed in Taiwanese strains (Fig. 5D, Supplemental Fig. S2D). With the addition of historic human P[19] strains and porcine strains sampled in 2014, two clusters (cluster I and II) were identified within the I5 genotype, each containing only Taiwanese strains^8^. Cluster I also clustered with porcine and porcine-like human strains found in other parts of the world, while cluster II strains have closest neighbors with one unusual Vietnamese G26P[19] strain and porcine strains from other Asian countries. The finding of I12 in the majority of our porcine and human RVA strains was unexpected, as the genotype was previously only found in rare G11P[25] and G5P[6] human strains but not in G9 porcine/human RVAs^26^,^27^,^28^. All strains harboring I12 gene grouped in a single common cluster. This cluster is most similar to an unusual Chinese G5P[6] human strain that was characterized as porcine-like^28^. In general, phylogeny of the genes encoding structural proteins of the Taiwanese G9 strains formed particular lineages distinct from those detected in other parts of the world.[Fig f6]

The NSP1 genes also showed variation as seen in the VP3 genes; however, the grouping of NSP1 and VP3 genes did not correlate with each other suggesting that the two genes evolved/reassorted independently (Fig. 5E, Supplemental Fig. S2E). The NSP1 genes of Taiwanese G9 strains could be classified into three clusters; cluster I consisted of the two G9P[19] human RVA strains and two porcine strains, and were within the same group as the Chinese porcine TM-a; cluster II was found to be a distinct Taiwan branch on the tree containing three porcine strains and the human G9P[13] strain that is distant from all other strains (85–89% identity); cluster III consisted of a single porcine G9P[23] strain that was related to the historic Gottfried porcine strain and the Indian porcine-like human mani-97 strain. The NSP2 genes were found in one distinct branch on the tree (Fig. 5F, Supplemental Fig. S2F); this branch was more closely related to porcine strains than to typical human strains. Similarly, the NSP3 genes exhibited almost the same clustering pattern except for the 2007 human G9P[19] strain, which was located on a distinct branch (Fig. 5G, Supplemental Fig. S2G). Of note is that all NSP3 genes were relatively closely related to human strains that may have been derived from a common ancestral strain that gave rise to the early human G9 strains 116E and DC5142. All but one strain had the E1 genotype for NSP4 that again clustered into a single lineage that is neighboring to but different from other human strains, while the G9P[13] human strain has a rare genotype, E9 (Fig. 5H, Supplemental Fig. S2H). Interestingly, the E9 genotype has never been found to be carried by human strains. Of interest, genotypes E1 and E9 cluster on a shared branch suggesting a single common ancestor for these two genotypes^29^,^30^,^31^. All NSP5 genes in Taiwanese G9 strains were of the H1 genotype and could be divided into two groups that differed in length and structure, although both were most closely related to porcine origin strains (Fig. 5I, Supplemental Fig. S2I). One group (group I) represented ‘normal’ NSP5 genes with an expected genome segment size (~660 bp); this group included four porcine strains and humans strains from 2007 and 2015. The other group (group II) exhibited partial duplication of the coding region sharing high similarity with each other and are structurally related to the Thai G9P[19] strain, Mc345, and the Belgian strains, 12R046 and BE2001, although phylogenetic analysis based on only the coding region (22~615 bp) showed that they are more similar to Mc345^19^,^21^,^32^. When compared with the rearranged NSP5 from Mc345, Taiwanese rearranged NSP5 does not have a 6 bp direct repeat sequence and its downstream CTG sequence at the site of duplication, rather, right after the first UAA stop codon there is a YTT sequence followed by the duplicated section starting from UGG copied from position 111 to 619 (Fig. 3)^32^. There are also many mutations in the duplicated sequence, sharing only 86% identity with the original sequence with over 60 substitution and exhibiting 3 or 4 deletions. It is of interest to note that of three G9P[19] strains (i.e. 2-1, 3-20, 4-1) isolated from the same Taiwanese farm only one strain (3-20) carried a normal NSP5, combined with genetic differences in the I and the A genotypes; this is consistent with the idea that several G9P[19] strains with backbone genes of different origins are circulating on the same farm. Similarly to the structural genes, in phylogenetic inference of the non-structural genes, the Taiwanese G9 strains typically formed genetic clusters that separated these strains from those detected in other parts of the world.[Fig f7]

Collectively, by using phylogenetic analysis, we found that nearly all genes of human G9 RVAs could be derived from one of the contemporary Taiwanese porcine RVA strains.

### Identification of host species specific motifs in the predicted protein sequences

**Figure 6 f6:**
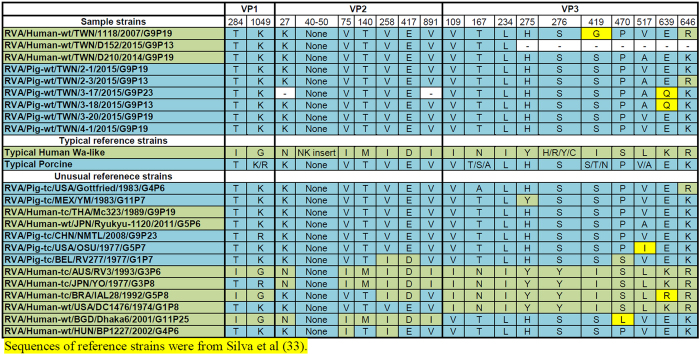
Amino acid variable positions of VP1, VP2, and VP3 in samples from this study and reference typical and unusual strains.

**Figure 7 f7:**
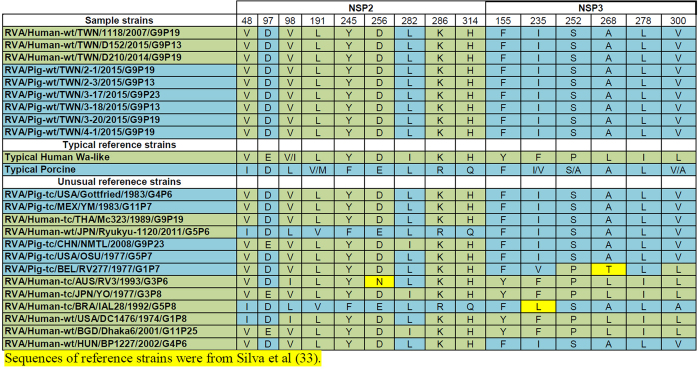
Amino acid variable positions of NSP2 and NSP3 in samples from this study and reference typical and unusual strains.

A recent study found that backbone genes of genotype 1 human and porcine strains can be distinguished by conserved patterns of amino acid substitutions within the VP1, VP2, VP3, NSP1, NSP2, and NSP3 proteins^33^. We performed analysis on these five proteins from the strains sampled and compared results with the reference strains sampled in the publication (Figs 6 and 7). All variable sites in the VP1, VP2, and NSP3 proteins were characteristic of typical porcine RVA. For VP3, while the D210 human strain and three strains from a pig farm in Pingtung (2-1, 3-20, 4-1) shared variable sites with porcine strains, two RVAs from a farm in Kaohsiung (3-17 and 3-18) had a unique glutamine rather than glutamic acid (at position 639). A strain from a different farm in Pingtung (2-3) and the human strain, 1118, had a change to aspartic acid in position 646 and for the human strain there is a further unique change at position 419 to glycine. All samples had NSP2 proteins that shared multiple conserved sites with Wa-like human origin NSP2 proteins but also had substitutions at positions 97 and 282 that resembled to patterns seen in porcine strains (D and L, respectively). Moreover, this combination of aa substitutions was detected in many reference human and porcine RVA strains^33^. Thus, the genesis of NSP2 is difficult to discern, as the particular variable site pattern found in this protein is shared among various porcine and human strains^33^.

## Conclusions

A new lineage of genotype G9 RVA strains emerged during the 1990 s, becoming the fifth most important genotype over time among human RVAs worldwide^12^,^34^. Other studies have reported additional genetic diversity of unusual and historic G9 RVA strains, not only in humans but also in pigs. In some instances the genetic relationship between porcine and human G9 strains provided evidence for interspecies transmission^15^,^21^. However, these previous sporadic human cases and animal infections have not been spatiotemporally linked, thus the epidemiological context of zoonotic transmissions in these reports was missing. Our study contrasts those earlier reports in term that phylogenetically closely related human and porcine G9 RVA strains were detected in the same rotavirus season from neighboring areas. Thus, the present survey provides conceivable scenario for direct transmission events of porcine G9 RVA strains to humans.

Due to the limited number of strains representing some of the currently known genetic variants of the G9 VP7 gene it is not possible to delineate the exact number of contemporary G9 lineages^12^. In addition, several lineages were detected only in historic strains from the 1980 s and no evidence suggests that these variants continue to circulate in any host species. It is currently unknown whether global distribution of the most common human G9 lineage may interfere with novel animal origin G9 strains to emerge in humans. In Taiwan the prevalence of common G9 RVA strains was around 20% over the past decade^25^. It is possible that widespread occurrence of these strains may not favor the local spread of novel G9 strains in human populations. It seems worth noting that the VP4 gene is directly associated with susceptibility and host species adaptation through mediating virus attachment to cells via host specific glycans and it still awaits formal demonstration whether genotypes P[13] or P[19] may contribute to the stable human-to-human transmissibility of the unique Taiwanese G9 strains^35^. Thus, continued surveillance is needed to detect whether the identified novel antigen combinations or a more favorable genotype constellation could facilitate the introduction of G9 strains in the general pediatric population in Taiwan or contribute notably to the diversity of globally dispersed RVA strains.

## Material and Methods

### Stool samples

A total of 2,444 human stool samples were collected in 2014 (n = 1,009) and 2015 (n = 1,435) from hospitalized children of less than 5 years old with symptoms of acute gastroenteritis (AGE) in hospital-based rotavirus surveillance conducted in 8 (2014) and 10 (2015) hospitals across Taiwan. AGE was defined as three or more episodes of either of these symptoms within 24 hours before presentation to hospital: watery diarrhea or loose stool, blood/mucus in stools, or vomiting. Stool specimens were sent to Taiwan CDC for bacterial and viral tests. Bacterial examinations included cultures for common enteropathogens, such as *Salmonella* species, *Shigella* species, *Vibrio* cholera, *Vibrio parahemolyticus*, pathogenic *E. coli*, and *Bacillus cereus*, etc., while viral tests included RT-PCR assays for norovirus and EIA screening of rotavirus as previously described^7^,^8^,^25^,^36^. Noroviruses GI and GII were identified by primer pairs G1SKF/R and G2SKF/R, respectively^37^.

For porcine samples, a total of 1,350 and 1,080 stool specimens were taken in 2014 and 2015, respectively, from various pig farms throughout Taiwan from nursing piglets (<1 month, 450 in 2014, 360 in 2015), weaned pigs (6 months, 450 in 2014, 360 in 2015), and mature pigs (>12 months, 450 in 2014, 360 in 2015) without information about diarrheal status. Viral and bacterial enteropathogenic agents were screened as above for human samples. All methods were carried out in accordance with relevant guidelines and regulations. All experimental protocols were approved by the institutional review boards of the Centers for Disease Control of Taiwan, each of the study hospitals sites and the National Health Research Institutes. During sample collection informed consent was obtained from all subjects. The study was approved by the institutional review boards of the Centers for Disease Control of Taiwan, each of the study hospitals sites and the National Health Research Institutes.

### RNA extraction

Viral RNA was extracted from 10% (wt/v) purified fecal supernatants by QIAmp Viral RNA Mini Kit (QIAGEN, Germany) or MagNA Pure LC DNA isolation kit (Roche Diagnostics GmbH, Germany) following the manufacturer’s instructions.

### Rotavirus detection and amplification of VP4, VP7, VP6, and NSP4 genes

Rotavirus genes were amplified from crude viral RNA extract by conventional two-step reverse-transcription-PCR (RT-PCR) with specific primer sets: VP4: 793 F/1583 R, con3/2, VP4F/R; VP7: 39 F/406 R/Beg9/End9, 9con1/2; VP6: GEN VP6F/R, VP6F/R, JRG7/8; NSP4: GEN NSP4F/R, JRG30/31. RNA was first mixed with 0.5 μl of each of the forward and reverse primers (10 μM) and denatured at 95 °C for 5 min and immediately chilled on ice. RT mixture was prepared with 2.5 μl of denatured dsRNA, 5 μl of water, 4 μl of 5X RT buffer, 6.4 μl of 2.5 mM dNTP, and 0.1 μl of AMV reverse transcriptase (New England Biolabs). The mixture was incubated at 42 °C for one hour. PCR was performed with the same set of primers (0.5 μl of 10 μM) with Blend Taq-Plus (Toyobo, Japan) according to manufacturer’s instructions. For 793 F/1583 R, con3/con2, VP4F/VP4R, VP6F/VP6Rand all NSP4 primer pairs, the following PCR program was used: an initial denaturation at 94 °C for 2 min followed by 40 cycles of 94 °C for 45 s, 52 °C for 45 s, and 72 °C for 1 min with a final extension at 72 °C for 7 min. For all VP7, GEN VP6F/GEN VP6R, and JRG7/JRG8 primer pairs, the following program was used: an initial denaturation at 94 °C for 2 min followed by 40 cycles of 94 °C for 45 s, 52 °C for 45 s, and 72 °C for 1 min and 30 s with a final extension at 72 °C for 10 min.

For EIA-positive samples that did not amplify with the above method, the following alternative RT protocol with SuperScript III (Thermo Fisher Scientific, MA, USA) reverse transcriptase was used. 1 μl of each of the forward and reverse primers were used instead of 0.5 μl to compensate for the presence of degenerate nucleotides. For dsRNA denaturation, 2.5 μl of RNA was mixed with 1.4 μl of DMSO, 1 μl of 10 mM dNTP, 1 μl of each of the forward and reverse primers, and 8 μl of water and heated at 95 °C for 5 min and chilled immediately on ice slurry. For the RT-PCR process, 4 μl of 5X RT buffer, 1 μl of 0.1 M DTT, and 0.1 μl of Superscript III reverse transcriptase was added to the denatured dsRNA mixture and incubated at 55 °C for one hour and 72 °C for 15 min for enzyme inactivation.

### Full rotavirus genome amplification

Two human G9 strains from 2014–2015 and six G9 porcine strains from 2015 were selected for full-length genome amplification. A historic G9 strain from 2007 previously described was also chosen for full genome sequencing^8^. For full genome amplification of 11 rotavirus dsRNA segments, the previously stated RT-PCR protocol with SuperScript III reverse transcriptase was used with the only exception that reverse transcription was carried out in the presence of a mixture of random hexamer oligonucleotides (300 μg/μl). A universal step-down PCR program was used for all amplicons with primer listed in Supplemental Table S1 online (0.5 μl of 10 μM): 94 °C for 2 min for initial denaturation, 4 cycles of 94 °C for 45 s, 70 °C for 45 s, and 72 °C for 1 min, 4 cycles of 94 °C for 45 s, 65 °C for 45 s, and 72 °C for 1 min; 4 cycles of 94 °C for 45 s, 70 °C for 45 s, and 72 °C for 1 min; 5 cycles of 94 °C for 45 s, 60 °C for 45 s, and 72 °C for 1 min; 5 cycles of 94 °C for 45 s, 55 °C for 45 °C, and 72 °C for 1 min; 30 cycles of 94 °C for 45 s, 50 °C for 45 s, and 72 °C for 1 min, and a final extension of 7 min at 72 °C. PCR products were visualized on a 1.5% agarose gel with ethidium bromide stain. For the segments that did not amplify with random primers, specific primers were used for both the RT and PCR processes. The PCR products were sequenced with their respective primers used in the amplification process.

The newly generated sequences were deposited in GenBank under the following accession numbers: KU739899 to KU740031.

### Genotyping and phylogenetic analysis

Sequences of each of the segments were assembled with Sequencher 4.5 (Gene Codes, MI). Genotypes were determined by comparing with available reference sequences using BLAST (www.ncbi.nlm.nih.gov/BLAST) and RotaC v2.0 genotyping tool (rotac.regatools.be)^38^. Sequences obtained from samples and reference sequences were aligned with MUSCLE and phylogenetic trees with 1,000 bootstraps replicates were created with neighbor-joining and maximum likehood methods with the best-fit substitution models determined by built-in model test application in MEGA6^39^. Variable amino acid positions of VP1, VP2, VP3, NSP1, NSP2, and NSP3 were identified by aligning protein sequence in MEGA6 and visually inspecting each of the variable sites that were identified as previously described^33^.

## Additional Information

**How to cite this article**: Wu, F.-T. *et al*. Novel G9 rotavirus strains co-circulate in children and pigs, Taiwan. *Sci. Rep.* **7**, 40731; doi: 10.1038/srep40731 (2017).

**Publisher's note:** Springer Nature remains neutral with regard to jurisdictional claims in published maps and institutional affiliations.

## Supplementary Material

Supplementary Information

## Figures and Tables

**Table 1:** Information of porcine (2014–2015) and human strains used in VP7 phylogenetic analysis.

Host	Strain	Date (year/month)	Age group	Location (region)	Location (County/city)	Rotavirus genotype	Sequenced genes
Porcine	2-1 (104-P-003-1-0189)	2015/02	Suckling	South	Pingtung County	G9P[19]	All 11
	2-3 (104-P-003-1-0215)	2015/02	Suckling	South	Pingtung County	G9P[13]	All 11
	3-17 (104-P-003-1-0334)	2015/03	Suckling	South	Kaohsiung City	G9P[23]	All 11
	3-18 (104-P-003-1-0335)	2015/03	Suckling	South	Kaohsiung City	G9P[13]	All 11
	3-20 (104-P-003-1-0370)	2015/03	Suckling	South	Pingtung County	G9P[19]	All 11
	4-1 (104-P-003-1-0487)	2015/04	Suckling	South	Pingtung County	G9P[19]	All 11
	103-P-003-1-1146	2014/10	Suckling	South	Chiayi County	G9P[19]	VP4, VP6, VP7
	103-P-003-1-1081	2014/10	Suckling	North	Miaoli County	G9P[23]	VP4, VP6, VP7
	103-P-004-1-0817	2014/08	Suckling	East	Hualien County	G9P[13]	VP4, VP6, VP7
	103-P-002-1-0511	2014/05	Suckling	Central	Nantou County	G9Px	VP4, VP6, VP7
	103-P-001-1-1322	2014/12	Suckling	North	Taoyuan City	G9P[13]	VP4, VP6, VP7
	103-P-002-1-0396	2014/04	Suckling	South	Yunlin County	G9P[23]	VP4, VP6, VP7
Human	07-96s-1118	2007/12	46 months	South	Kaohsiung City	G9P[19]	All 11
	103-701-D210	2014/12	28 months	South	Kaohsiung City	G9P[19]	All 11
	104-701-D152	2015/4	14 months	South	Pingtung County	G9P[13]	All 11 (VP3 and VP4 partial)
