# Supplementary material for: Novel G9 rotavirus strains co-circulate in children and pigs, Taiwan
Source: Sci Rep. 2017 Jan 18;7:40731. doi: 10.1038/srep40731 (PMC5241653; doi:10.1038/srep40731)
Supplement: Supplementary Information [file srep40731-s1.pdf]

Supplementary Information

Novel G9 rotavirus strains co-circulate in children and pigs, Taiwan

Fang-Tzy Wu[1*], Krisztián Bányai[2*], Baoming Jiang[3], Luke Tzu-Chi Liu[1], Szilvia Marton[2], Yhu-Chering Huang[4], Li-Min Huang[5], Liao Ming-Hui [6#], Chao A. Hsiung[7#]

[1] Center for Diagnostics and Vaccine Development, Centers for Disease Control, Taipei, Taiwan

[2] Institute for Veterinary Medical Research, Centre for Agricultural Research, Hungarian Academy of Sciences, Budapest, Hungary

[3] Centers for Disease Control and Prevention, Atlanta, Georgia, USA

[4] Department of Pediatrics, Chang Gung Children's Hospital, Chang Gung Memorial Hospital, Chang Gung University, College of Medicine, Taoyuan, Taiwan

[5] Department of Pediatrics, National Taiwan University Hospital, College of Medicine, National Taiwan University, Taipei, Taiwan

[6] College of Veterinary Medicine, National Pingtung University of Science Technology, Taiwan

[7] Institute of Population Health Sciences, National Health Research Institutes, Zhunan, Taiwan

* # These authors have equally contributed to this work.

Supplementary Figure S1. Close-up map of part of Kaohsiung city and Pingtung county. Strain names (in red) are marked in parentheses adjacent to names of district/township from which G9 positive pig farms and residences of patients are located. Map modified from Google Maps: Map data ©2015 Google, ZENRIN, with Adobe Photoshop CS 8.0 (www.photoshop.com)

Legend:

Red squares: 2015 porcine VP7 sublineage I strains
Red circle: 2014 Human VP7 sublineage I strain
Blue triangles: 2014 porcine VP7 sublineage II strains
Blue circles: 2007 and 2015 Human VP7 sublineage II strains
Blue squares: 2015 porcine VP7 sublineage II strains

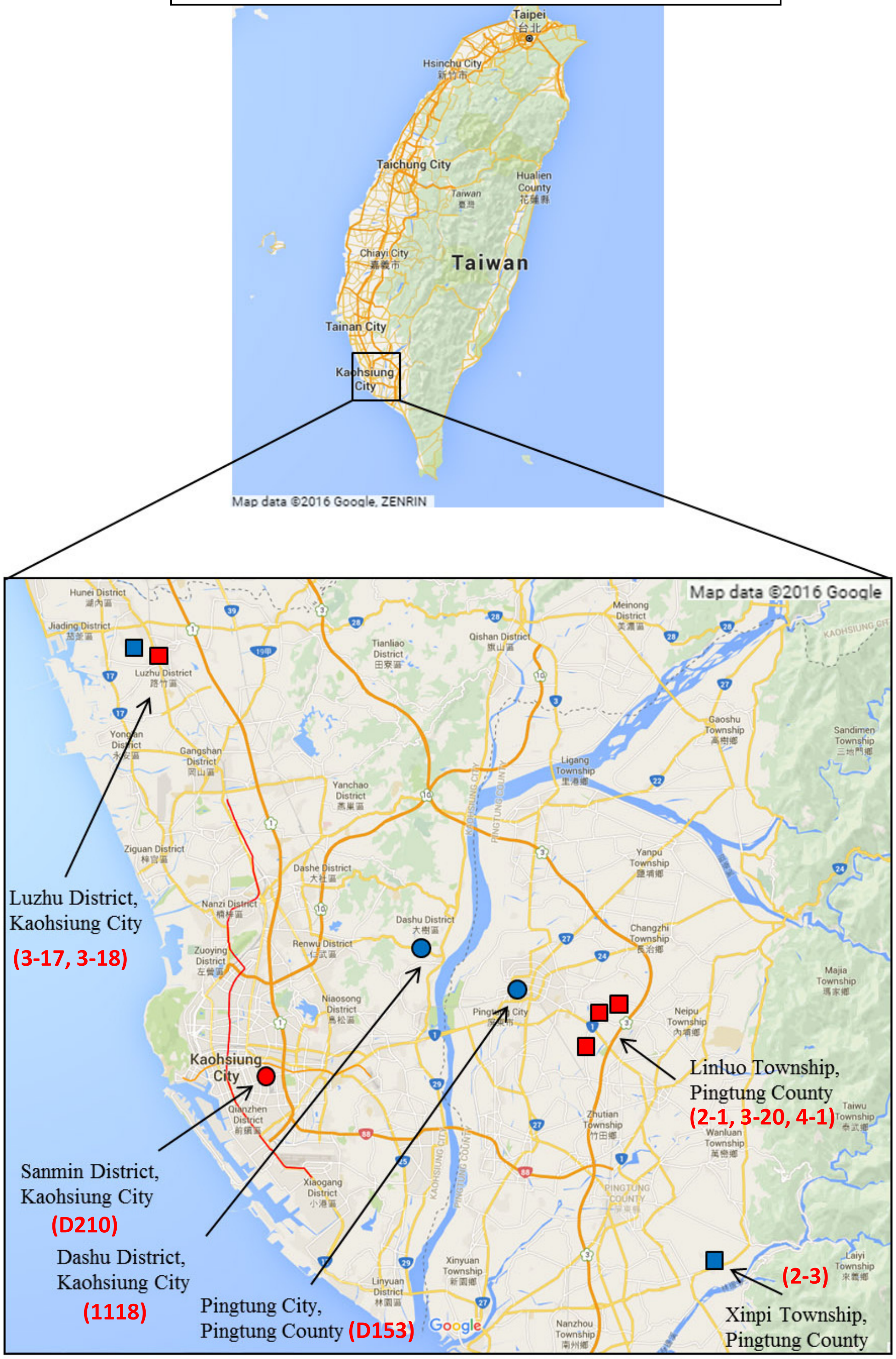

Supplementary Figure S2. Extended phylogenetic trees of the simplified trees in Figure 4 (inserted main text). (A) VP1, (B) VP2, (C) VP3, (D) VP6, (E) NSP1, (F) NSP2, (G) NSP3, (H) NSP4, and (I) NSP5. Refer to Fig. 4 for explanation of methods used and legends. MEGA6 was used to generate neighbor-joining (maximum composite likelihood) or maximum likelihood trees with 1,000 bootstrap replicates using the best-fit substitution models determined by the built-in model test application: GTR=General Time Reversible, T92=Tamura 92, HKY=Hasegawa-Kishino-Yano 85, TN93=Tamura-Nei 93, G=gamme sites, I=invariant sites. The region within each of the gene segment used for each analysis is also shown. Open circles indicate Taiwanese human strains, closed circles denote Taiwanese porcine strains from 2015, open diamonds denote Taiwanese porcine strains from 2014.

A. VP1
GTR+G+I
Positions 19-3270 bp

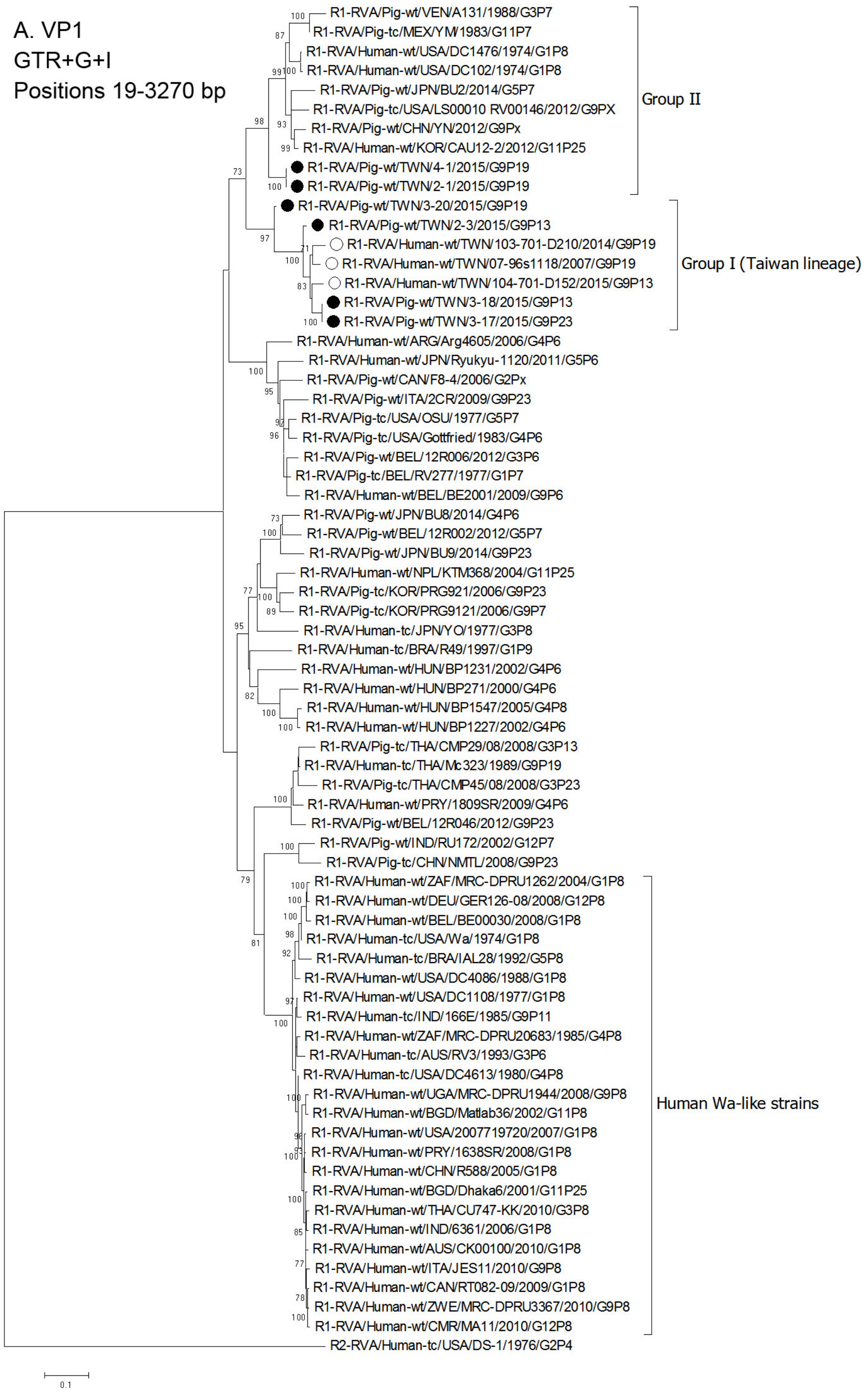

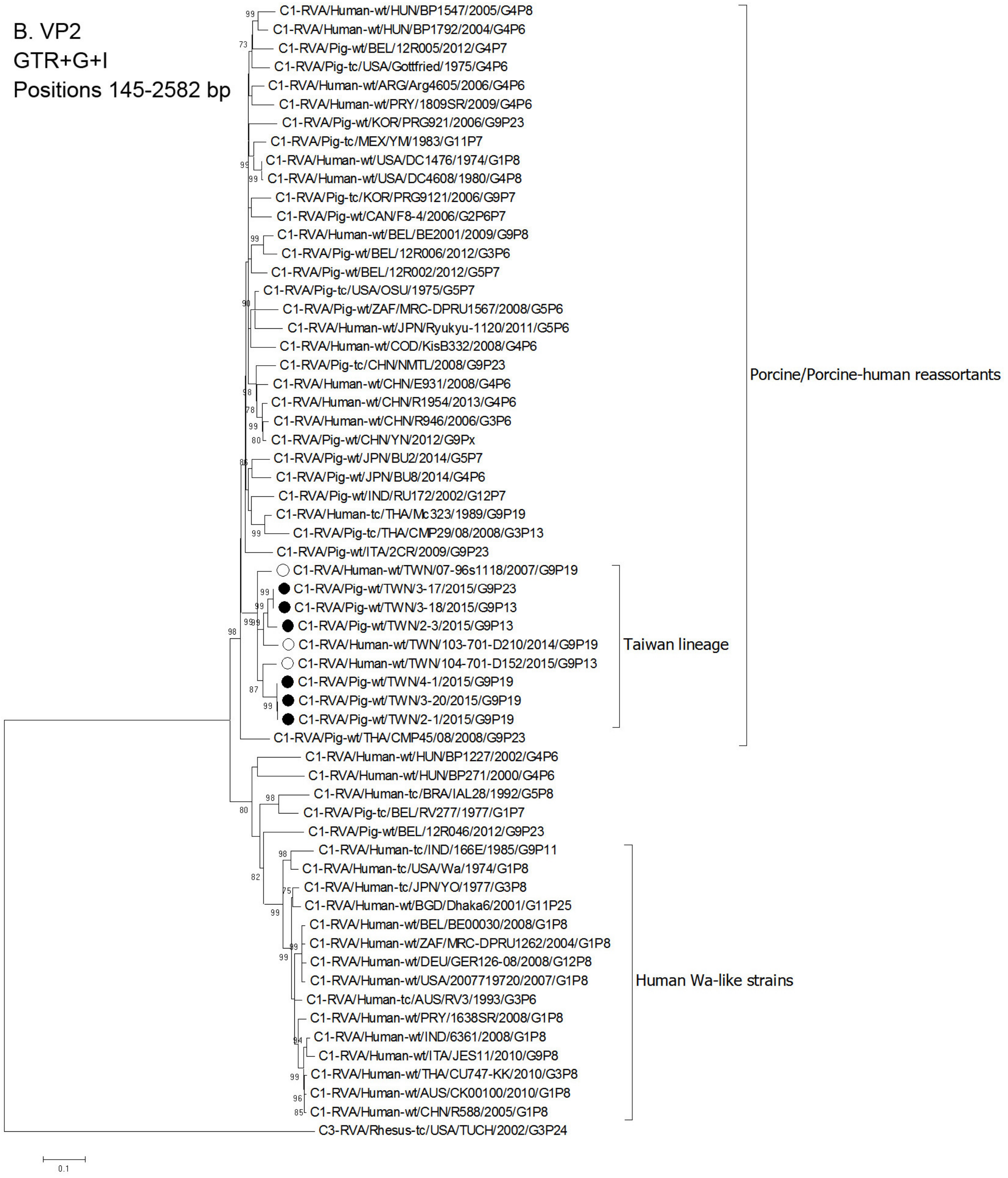
B. VP2
GTR+G+I
Positions 145-2582 bp
C1-RVA/Human-wt/HUN/BP1547/2005/G4P8
C1-RVA/Human-wt/HUN/BP1792/2004/G4P6
C1-RVA/Pig-wt/BEL/12R005/2012/G4P7
C1-RVA/Pig-tc/USA/Gottfried/1975/G4P6
C1-RVA/Human-wt/ARG/Arg4605/2006/G4P6
C1-RVA/Human-wt/PRY/1809SR/2009/G4P6
C1-RVA/Pig-wt/KOR/PRG921/2006/G9P23
C1-RVA/Pig-tc/MEX/YM/1983/G11P7
C1-RVA/Human-wt/USA/DC1476/1974/G1P8
C1-RVA/Human-wt/USA/DC4608/1980/G4P8
C1-RVA/Pig-tc/KOR/PRG9121/2006/G9P7
C1-RVA/Pig-wt/CAN/F8-4/2006/G2P6P7
C1-RVA/Human-wt/BEL/BE2001/2009/G9P8
C1-RVA/Pig-wt/BEL/12R006/2012/G3P6
C1-RVA/Pig-wt/BEL/12R002/2012/G5P7
C1-RVA/Pig-tc/USA/OSU/1975/G5P7
C1-RVA/Pig-wt/ZAF/MRC-DPRU1567/2008/G5P6
C1-RVA/Human-wt/JPN/Ryukyu-1120/2011/G5P6
C1-RVA/Human-wt/COD/KisB332/2008/G4P6
C1-RVA/Pig-tc/CHN/NMTL/2008/G9P23
C1-RVA/Human-wt/CHN/E931/2008/G4P6
C1-RVA/Human-wt/CHN/R1954/2013/G4P6
C1-RVA/Human-wt/CHN/R946/2006/G3P6
C1-RVA/Pig-wt/CHN/YN/2012/G9Px
C1-RVA/Pig-wt/JPN/BU2/2014/G5P7
C1-RVA/Pig-wt/JPN/BU8/2014/G4P6
C1-RVA/Pig-wt/IND/RU172/2002/G12P7
C1-RVA/Human-tc/THA/Mc323/1989/G9P19
C1-RVA/Pig-tc/THA/CMP29/08/2008/G3P13
C1-RVA/Pig-wt/ITA/2CR/2009/G9P23
C1-RVA/Human-wt/TWN/07-96s1118/2007/G9P19
C1-RVA/Pig-wt/TWN/3-17/2015/G9P23
C1-RVA/Pig-wt/TWN/3-18/2015/G9P13
C1-RVA/Pig-wt/TWN/2-3/2015/G9P13
C1-RVA/Human-wt/TWN/103-701-D210/2014/G9P19
C1-RVA/Human-wt/TWN/104-701-D152/2015/G9P13
C1-RVA/Pig-wt/TWN/4-1/2015/G9P19
C1-RVA/Pig-wt/TWN/3-20/2015/G9P19
C1-RVA/Pig-wt/TWN/2-1/2015/G9P19
C1-RVA/Pig-wt/THA/CMP45/08/2008/G9P23
C1-RVA/Human-wt/HUN/BP1227/2002/G4P6
C1-RVA/Human-wt/HUN/BP271/2000/G4P6
C1-RVA/Human-tc/BRA/IAL28/1992/G5P8
C1-RVA/Pig-tc/BEL/RV277/1977/G1P7
C1-RVA/Pig-wt/BEL/12R046/2012/G9P23
C1-RVA/Human-tc/IND/166E/1985/G9P11
C1-RVA/Human-tc/USA/Wa/1974/G1P8
C1-RVA/Human-tc/JPN/YO/1977/G3P8
C1-RVA/Human-wt/BGD/Dhaka6/2001/G11P25
C1-RVA/Human-wt/BEL/BE00030/2008/G1P8
C1-RVA/Human-wt/ZAF/MRC-DPRU1262/2004/G1P8
C1-RVA/Human-wt/DEU/GER126-08/2008/G12P8
C1-RVA/Human-wt/USA/2007719720/2007/G1P8
C1-RVA/Human-tc/AUS/RV3/1993/G3P6
C1-RVA/Human-wt/PRY/1638SR/2008/G1P8
C1-RVA/Human-wt/IND/6361/2008/G1P8
C1-RVA/Human-wt/ITA/JES11/2010/G9P8
C1-RVA/Human-wt/THA/CU747-KK/2010/G3P8
C1-RVA/Human-wt/AUS/CK00100/2010/G1P8
C1-RVA/Human-wt/CHN/R588/2005/G1P8
C3-RVA/Rhesus-tc/USA/TUCH/2002/G3P24
Porcine/Porcine-human reassortants
Taiwan lineage
Human Wa-like strains
0.1

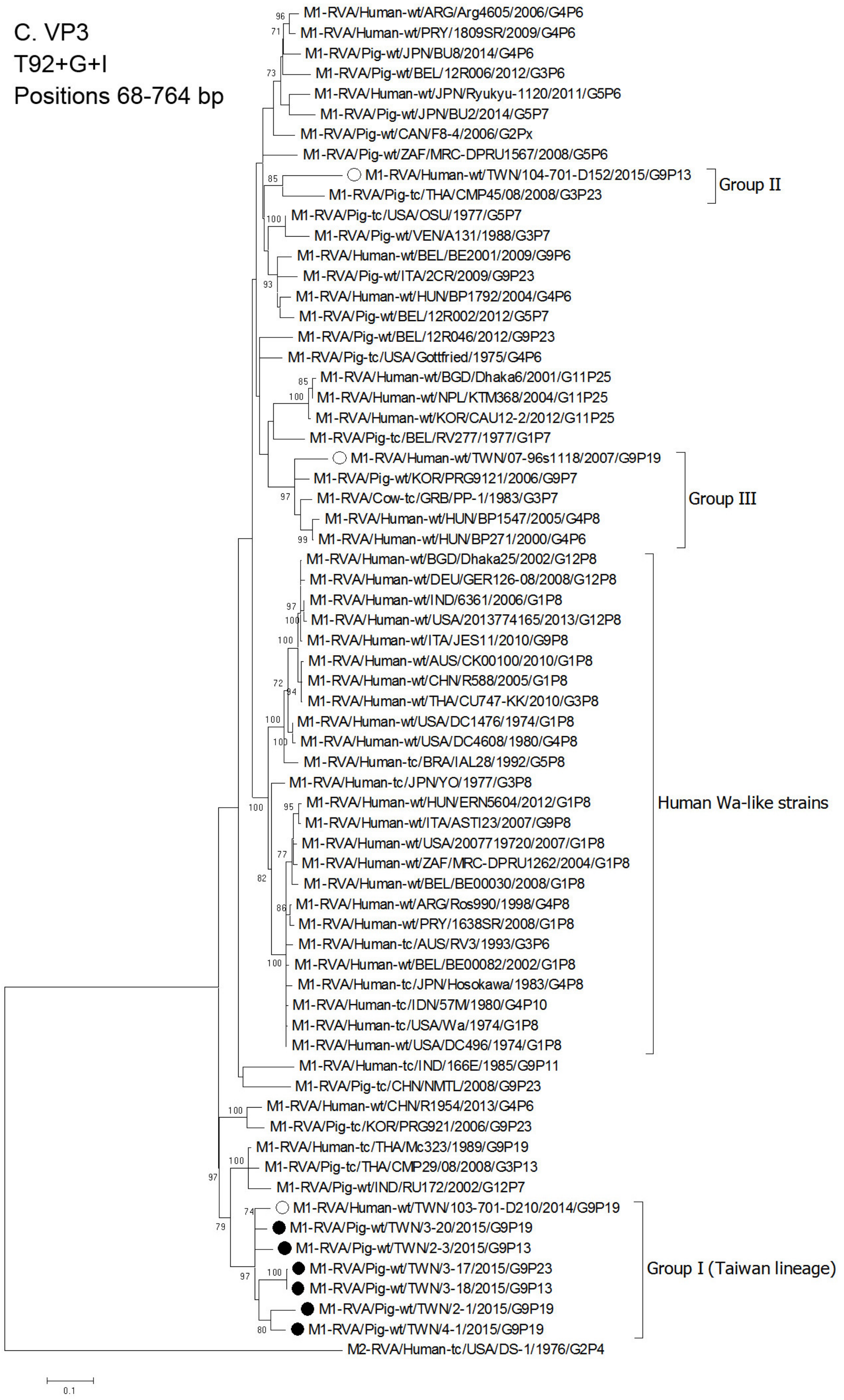
C. VP3
T92+G+I
Positions 68-764 bp
M1-RVA/Human-wt/ARG/Arg4605/2006/G4P6
M1-RVA/Human-wt/PRY/1809SR/2009/G4P6
M1-RVA/Pig-wt/JPN/BU8/2014/G4P6
M1-RVA/Pig-wt/BEL/12R006/2012/G3P6
M1-RVA/Human-wt/JPN/Ryukyu-1120/2011/G5P6
M1-RVA/Pig-wt/JPN/BU2/2014/G5P7
M1-RVA/Pig-wt/CAN/F8-4/2006/G2Px
M1-RVA/Pig-wt/ZAF/MRC-DPRU1567/2008/G5P6
M1-RVA/Human-wt/TWN/104-701-D152/2015/G9P13
M1-RVA/Pig-tc/THA/CMP45/08/2008/G3P23
Group II
M1-RVA/Pig-tc/USA/OSU/1977/G5P7
M1-RVA/Pig-wt/VEN/A131/1988/G3P7
M1-RVA/Human-wt/BEL/BE2001/2009/G9P6
M1-RVA/Pig-wt/ITA/2CR/2009/G9P23
M1-RVA/Human-wt/HUN/BP1792/2004/G4P6
M1-RVA/Pig-wt/BEL/12R002/2012/G5P7
M1-RVA/Pig-wt/BEL/12R046/2012/G9P23
M1-RVA/Pig-tc/USA/Gottfried/1975/G4P6
M1-RVA/Human-wt/BGD/Dhaka6/2001/G11P25
M1-RVA/Human-wt/NPL/KTM368/2004/G11P25
M1-RVA/Human-wt/KOR/CAU12-2/2012/G11P25
M1-RVA/Pig-tc/BEL/RV277/1977/G1P7
M1-RVA/Human-wt/TWN/07-96s1118/2007/G9P19
M1-RVA/Pig-wt/KOR/PRG9121/2006/G9P7
M1-RVA/Cow-tc/GRB/PP-1/1983/G3P7
M1-RVA/Human-wt/HUN/BP1547/2005/G4P8
M1-RVA/Human-wt/HUN/BP271/2000/G4P6
Group III
M1-RVA/Human-wt/BGD/Dhaka25/2002/G12P8
M1-RVA/Human-wt/DEU/GER126-08/2008/G12P8
M1-RVA/Human-wt/IND/6361/2006/G1P8
M1-RVA/Human-wt/USA/2013774165/2013/G12P8
M1-RVA/Human-wt/ITA/JES11/2010/G9P8
M1-RVA/Human-wt/AUS/CK00100/2010/G1P8
M1-RVA/Human-wt/CHN/R588/2005/G1P8
M1-RVA/Human-wt/THA/CU747-KK/2010/G3P8
M1-RVA/Human-wt/USA/DC1476/1974/G1P8
M1-RVA/Human-wt/USA/DC4608/1980/G4P8
M1-RVA/Human-tc/BRA/IAL28/1992/G5P8
M1-RVA/Human-tc/JPN/YO/1977/G3P8
M1-RVA/Human-wt/HUN/ERN5604/2012/G1P8
M1-RVA/Human-wt/ITA/ASTI23/2007/G9P8
M1-RVA/Human-wt/USA/2007719720/2007/G1P8
M1-RVA/Human-wt/ZAF/MRC-DPRU1262/2004/G1P8
M1-RVA/Human-wt/BEL/BE00030/2008/G1P8
M1-RVA/Human-wt/ARG/Ros990/1998/G4P8
M1-RVA/Human-wt/PRY/1638SR/2008/G1P8
M1-RVA/Human-tc/AUS/RV3/1993/G3P6
M1-RVA/Human-wt/BEL/BE00082/2002/G1P8
M1-RVA/Human-tc/JPN/Hosokawa/1983/G4P8
M1-RVA/Human-tc/IDN/57M/1980/G4P10
M1-RVA/Human-tc/USA/Wa/1974/G1P8
M1-RVA/Human-wt/USA/DC496/1974/G1P8
Human Wa-like strains
M1-RVA/Human-tc/IND/166E/1985/G9P11
M1-RVA/Pig-tc/CHN/NMTL/2008/G9P23
M1-RVA/Human-wt/CHN/R1954/2013/G4P6
M1-RVA/Pig-tc/KOR/PRG921/2006/G9P23
M1-RVA/Human-tc/THA/Mc323/1989/G9P19
M1-RVA/Pig-tc/THA/CMP29/08/2008/G3P13
M1-RVA/Pig-wt/IND/RU172/2002/G12P7
M1-RVA/Human-wt/TWN/103-701-D210/2014/G9P19
M1-RVA/Pig-wt/TWN/3-20/2015/G9P19
M1-RVA/Pig-wt/TWN/2-3/2015/G9P13
M1-RVA/Pig-wt/TWN/3-17/2015/G9P23
M1-RVA/Pig-wt/TWN/3-18/2015/G9P13
M1-RVA/Pig-wt/TWN/2-1/2015/G9P19
M1-RVA/Pig-wt/TWN/4-1/2015/G9P19
Group I (Taiwan lineage)
M2-RVA/Human-tc/USA/DS-1/1976/G2P4
0.1

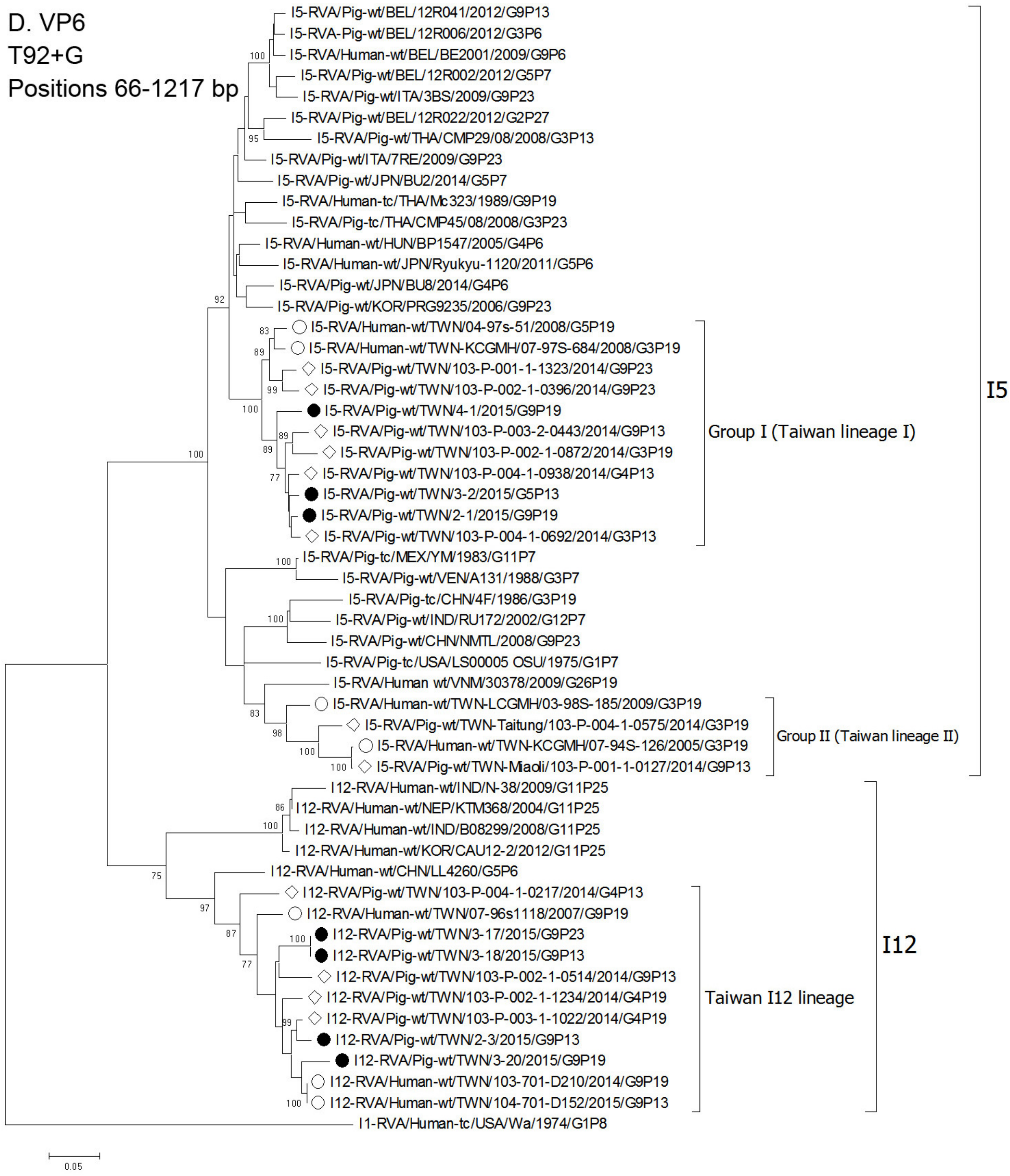
D. VP6
T92+G
Positions 66-1217 bp
I5-RVA/Pig-wt/BEL/12R041/2012/G9P13
I5-RVA-Pig-wt/BEL/12R006/2012/G3P6
I5-RVA/Human-wt/BEL/BE2001/2009/G9P6
I5-RVA/Pig-wt/BEL/12R002/2012/G5P7
I5-RVA/Pig-wt/ITA/3BS/2009/G9P23
I5-RVA/Pig-wt/BEL/12R022/2012/G2P27
I5-RVA/Pig-wt/THA/CMP29/08/2008/G3P13
I5-RVA/Pig-wt/ITA/7RE/2009/G9P23
I5-RVA/Pig-wt/JPN/BU2/2014/G5P7
I5-RVA/Human-tc/THA/Mc323/1989/G9P19
I5-RVA/Pig-tc/THA/CMP45/08/2008/G3P23
I5-RVA/Human-wt/HUN/BP1547/2005/G4P6
I5-RVA/Human-wt/JPN/Ryukyu-1120/2011/G5P6
I5-RVA/Pig-wt/JPN/BU8/2014/G4P6
I5-RVA/Pig-wt/KOR/PRG9235/2006/G9P23
I5-RVA/Human-wt/TWN/04-97s-51/2008/G5P19
I5-RVA/Human-wt/TWN-KCGMH/07-97S-684/2008/G3P19
I5-RVA/Pig-wt/TWN/103-P-001-1-1323/2014/G9P23
I5-RVA/Pig-wt/TWN/103-P-002-1-0396/2014/G9P23
I5-RVA/Pig-wt/TWN/4-1/2015/G9P19
I5-RVA/Pig-wt/TWN/103-P-003-2-0443/2014/G9P13
I5-RVA/Pig-wt/TWN/103-P-002-1-0872/2014/G3P19
I5-RVA/Pig-wt/TWN/103-P-004-1-0938/2014/G4P13
I5-RVA/Pig-wt/TWN/3-2/2015/G5P13
I5-RVA/Pig-wt/TWN/2-1/2015/G9P19
I5-RVA/Pig-wt/TWN/103-P-004-1-0692/2014/G3P13
Group I (Taiwan lineage I)
I5-RVA/Pig-tc/MEX/YM/1983/G11P7
I5-RVA/Pig-wt/VEN/A131/1988/G3P7
I5-RVA/Pig-tc/CHN/4F/1986/G3P19
I5-RVA/Pig-wt/IND/RU172/2002/G12P7
I5-RVA/Pig-wt/CHN/NMTL/2008/G9P23
I5-RVA/Pig-tc/USA/LS00005 OSU/1975/G1P7
I5-RVA/Human wt/VNM/30378/2009/G26P19
I5-RVA/Human-wt/TWN-LCGMH/03-98S-185/2009/G3P19
I5-RVA/Pig-wt/TWN-Taitung/103-P-004-1-0575/2014/G3P19
I5-RVA/Human-wt/TWN-KCGMH/07-94S-126/2005/G3P19
I5-RVA/Pig-wt/TWN-Miaoli/103-P-001-1-0127/2014/G9P13
Group II (Taiwan lineage II)
I5
I12-RVA/Human-wt/IND/N-38/2009/G11P25
I12-RVA/Human-wt/NEP/KTM368/2004/G11P25
I12-RVA/Human-wt/IND/B08299/2008/G11P25
I12-RVA/Human-wt/KOR/CAU12-2/2012/G11P25
I12-RVA/Human-wt/CHN/LL4260/G5P6
I12-RVA/Pig-wt/TWN/103-P-004-1-0217/2014/G4P13
I12-RVA/Human-wt/TWN/07-96s1118/2007/G9P19
I12-RVA/Pig-wt/TWN/3-17/2015/G9P23
I12-RVA/Pig-wt/TWN/3-18/2015/G9P13
I12-RVA/Pig-wt/TWN/103-P-002-1-0514/2014/G9P13
I12-RVA/Pig-wt/TWN/103-P-002-1-1234/2014/G4P19
I12-RVA/Pig-wt/TWN/103-P-003-1-1022/2014/G4P19
I12-RVA/Pig-wt/TWN/2-3/2015/G9P13
I12-RVA/Pig-wt/TWN/3-20/2015/G9P19
I12-RVA/Human-wt/TWN/103-701-D210/2014/G9P19
I12-RVA/Human-wt/TWN/104-701-D152/2015/G9P13
Taiwan I12 lineage
I12
I1-RVA/Human-tc/USA/Wa/1974/G1P8
0.05

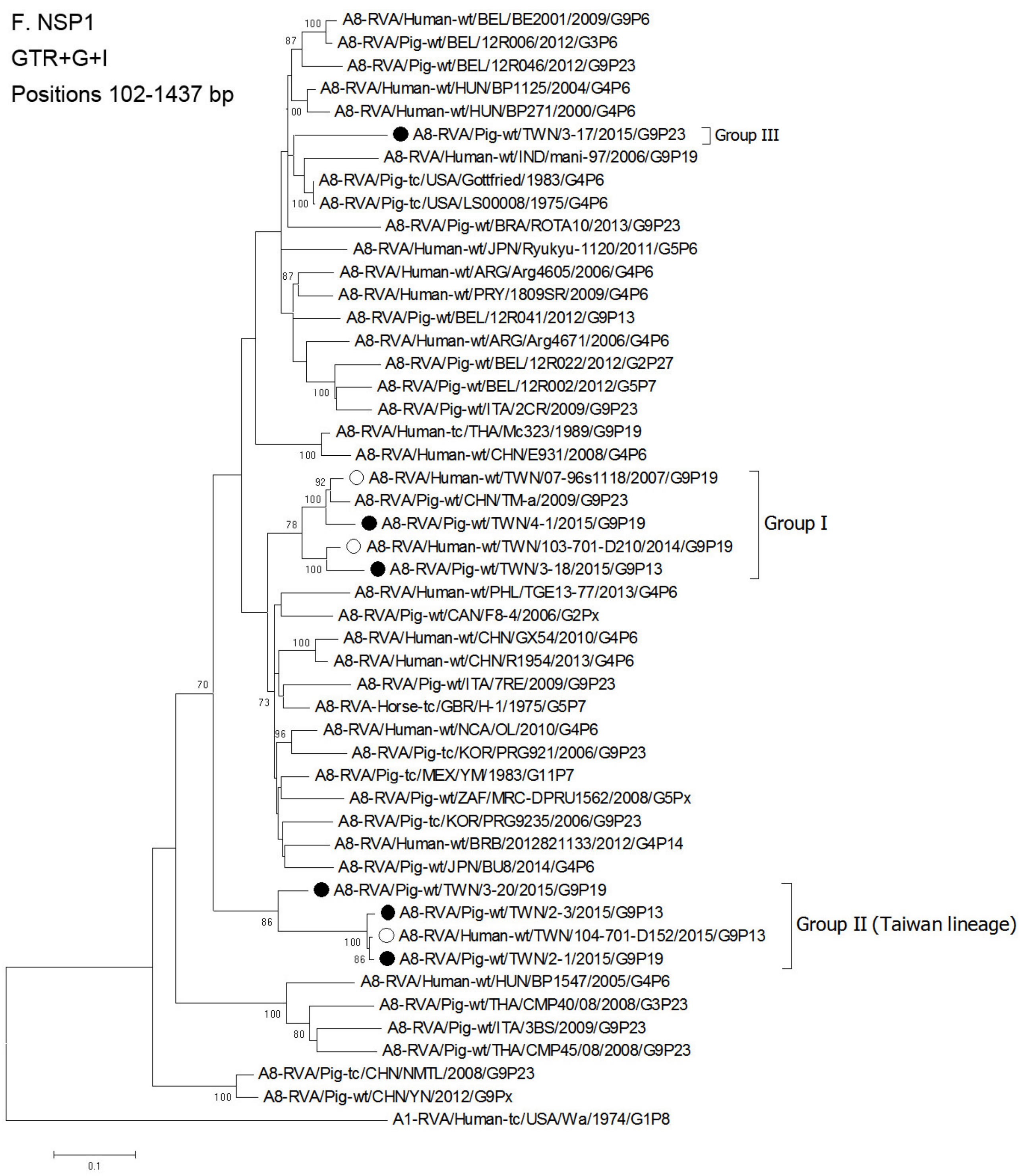
F. NSP1
GTR+G+I
Positions 102-1437 bp
A8-RVA/Human-wt/BEL/BE2001/2009/G9P6
A8-RVA/Pig-wt/BEL/12R006/2012/G3P6
A8-RVA/Pig-wt/BEL/12R046/2012/G9P23
A8-RVA/Human-wt/HUN/BP1125/2004/G4P6
A8-RVA/Human-wt/HUN/BP271/2000/G4P6
A8-RVA/Pig-wt/TWN/3-17/2015/G9P23
Group III
A8-RVA/Human-wt/IND/mani-97/2006/G9P19
A8-RVA/Pig-tc/USA/Gottfried/1983/G4P6
A8-RVA/Pig-tc/USA/LS00008/1975/G4P6
A8-RVA/Pig-wt/BRA/ROTA10/2013/G9P23
A8-RVA/Human-wt/JPN/Ryukyu-1120/2011/G5P6
A8-RVA/Human-wt/ARG/Arg4605/2006/G4P6
A8-RVA/Human-wt/PRY/1809SR/2009/G4P6
A8-RVA/Pig-wt/BEL/12R041/2012/G9P13
A8-RVA/Human-wt/ARG/Arg4671/2006/G4P6
A8-RVA/Pig-wt/BEL/12R022/2012/G2P27
A8-RVA/Pig-wt/BEL/12R002/2012/G5P7
A8-RVA/Pig-wt/ITA/2CR/2009/G9P23
A8-RVA/Human-tc/THA/Mc323/1989/G9P19
A8-RVA/Human-wt/CHN/E931/2008/G4P6
A8-RVA/Human-wt/TWN/07-96s1118/2007/G9P19
A8-RVA/Pig-wt/CHN/TM-a/2009/G9P23
A8-RVA/Pig-wt/TWN/4-1/2015/G9P19
A8-RVA/Human-wt/TWN/103-701-D210/2014/G9P19
A8-RVA/Pig-wt/TWN/3-18/2015/G9P13
Group I
A8-RVA/Human-wt/PHL/TGE13-77/2013/G4P6
A8-RVA/Pig-wt/CAN/F8-4/2006/G2Px
A8-RVA/Human-wt/CHN/GX54/2010/G4P6
A8-RVA/Human-wt/CHN/R1954/2013/G4P6
A8-RVA/Pig-wt/ITA/7RE/2009/G9P23
A8-RVA-Horse-tc/GBR/H-1/1975/G5P7
A8-RVA/Human-wt/NCA/OL/2010/G4P6
A8-RVA/Pig-tc/KOR/PRG921/2006/G9P23
A8-RVA/Pig-tc/MEX/YM/1983/G11P7
A8-RVA/Pig-wt/ZAF/MRC-DPRU1562/2008/G5Px
A8-RVA/Pig-tc/KOR/PRG9235/2006/G9P23
A8-RVA/Human-wt/BRB/2012821133/2012/G4P14
A8-RVA/Pig-wt/JPN/BU8/2014/G4P6
A8-RVA/Pig-wt/TWN/3-20/2015/G9P19
A8-RVA/Pig-wt/TWN/2-3/2015/G9P13
A8-RVA/Human-wt/TWN/104-701-D152/2015/G9P13
A8-RVA/Pig-wt/TWN/2-1/2015/G9P19
Group II (Taiwan lineage)
A8-RVA/Human-wt/HUN/BP1547/2005/G4P6
A8-RVA/Pig-wt/THA/CMP40/08/2008/G3P23
A8-RVA/Pig-wt/ITA/3BS/2009/G9P23
A8-RVA/Pig-wt/THA/CMP45/08/2008/G9P23
A8-RVA/Pig-tc/CHN/NMTL/2008/G9P23
A8-RVA/Pig-wt/CHN/YN/2012/G9Px
A1-RVA/Human-tc/USA/Wa/1974/G1P8
0.1

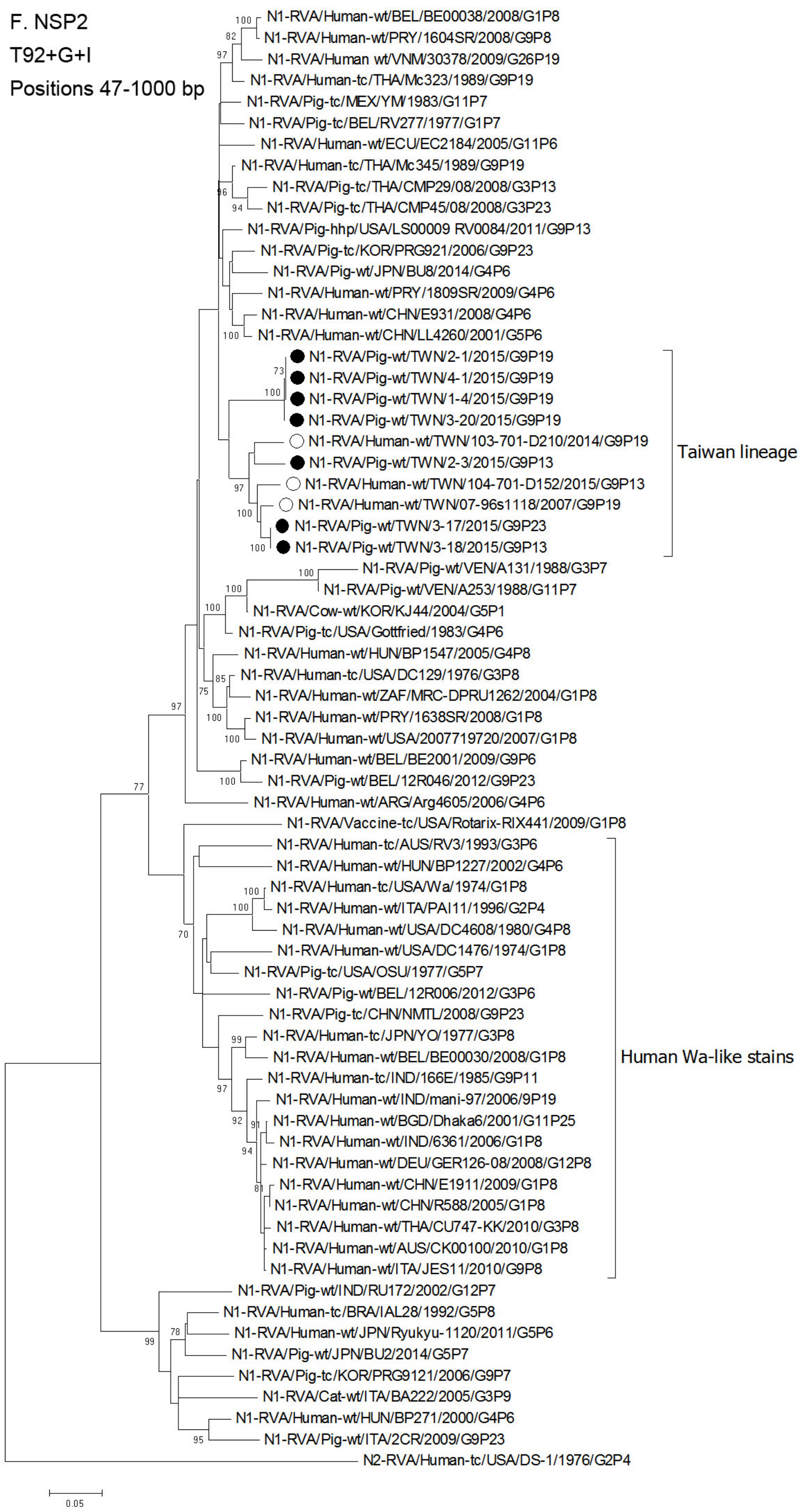
F. NSP2
T92+G+I
Positions 47-1000 bp
N1-RVA/Human-wt/BEL/BE00038/2008/G1P8
N1-RVA/Human-wt/PRY/1604SR/2008/G9P8
N1-RVA/Human wt/VNM/30378/2009/G26P19
N1-RVA/Human-tc/THA/Mc323/1989/G9P19
N1-RVA/Pig-tc/MEX/YM/1983/G11P7
N1-RVA/Pig-tc/BEL/RV277/1977/G1P7
N1-RVA/Human-wt/ECU/EC2184/2005/G11P6
N1-RVA/Human-tc/THA/Mc345/1989/G9P19
N1-RVA/Pig-tc/THA/CMP29/08/2008/G3P13
N1-RVA/Pig-tc/THA/CMP45/08/2008/G3P23
N1-RVA/Pig-hhp/USA/LS00009 RV0084/2011/G9P13
N1-RVA/Pig-tc/KOR/PRG921/2006/G9P23
N1-RVA/Pig-wt/JPN/BU8/2014/G4P6
N1-RVA/Human-wt/PRY/1809SR/2009/G4P6
N1-RVA/Human-wt/CHN/E931/2008/G4P6
N1-RVA/Human-wt/CHN/LL4260/2001/G5P6
N1-RVA/Pig-wt/TWN/2-1/2015/G9P19
N1-RVA/Pig-wt/TWN/4-1/2015/G9P19
N1-RVA/Pig-wt/TWN/1-4/2015/G9P19
N1-RVA/Pig-wt/TWN/3-20/2015/G9P19
N1-RVA/Human-wt/TWN/103-701-D210/2014/G9P19
N1-RVA/Pig-wt/TWN/2-3/2015/G9P13
N1-RVA/Human-wt/TWN/104-701-D152/2015/G9P13
N1-RVA/Human-wt/TWN/07-96s1118/2007/G9P19
N1-RVA/Pig-wt/TWN/3-17/2015/G9P23
N1-RVA/Pig-wt/TWN/3-18/2015/G9P13
Taiwan lineage
N1-RVA/Pig-wt/VEN/A131/1988/G3P7
N1-RVA/Pig-wt/VEN/A253/1988/G11P7
N1-RVA/Cow-wt/KOR/KJ44/2004/G5P1
N1-RVA/Pig-tc/USA/Gottfried/1983/G4P6
N1-RVA/Human-wt/HUN/BP1547/2005/G4P8
N1-RVA/Human-tc/USA/DC129/1976/G3P8
N1-RVA/Human-wt/ZAF/MRC-DPRU1262/2004/G1P8
N1-RVA/Human-wt/PRY/1638SR/2008/G1P8
N1-RVA/Human-wt/USA/2007719720/2007/G1P8
N1-RVA/Human-wt/BEL/BE2001/2009/G9P6
N1-RVA/Pig-wt/BEL/12R046/2012/G9P23
N1-RVA/Human-wt/ARG/Arg4605/2006/G4P6
N1-RVA/Vaccine-tc/USA/Rotarix-RIX441/2009/G1P8
N1-RVA/Human-tc/AUS/RV3/1993/G3P6
N1-RVA/Human-wt/HUN/BP1227/2002/G4P6
N1-RVA/Human-tc/USA/Wa/1974/G1P8
N1-RVA/Human-wt/ITA/PAI11/1996/G2P4
N1-RVA/Human-wt/USA/DC4608/1980/G4P8
N1-RVA/Human-wt/USA/DC1476/1974/G1P8
N1-RVA/Pig-tc/USA/OSU/1977/G5P7
N1-RVA/Pig-wt/BEL/12R006/2012/G3P6
N1-RVA/Pig-tc/CHN/NMTL/2008/G9P23
N1-RVA/Human-tc/JPN/YO/1977/G3P8
N1-RVA/Human-wt/BEL/BE00030/2008/G1P8
N1-RVA/Human-tc/IND/166E/1985/G9P11
N1-RVA/Human-wt/IND/mani-97/2006/9P19
N1-RVA/Human-wt/BGD/Dhaka6/2001/G11P25
N1-RVA/Human-wt/IND/6361/2006/G1P8
N1-RVA/Human-wt/DEU/GER126-08/2008/G12P8
N1-RVA/Human-wt/CHN/E1911/2009/G1P8
N1-RVA/Human-wt/CHN/R588/2005/G1P8
N1-RVA/Human-wt/THA/CU747-KK/2010/G3P8
N1-RVA/Human-wt/AUS/CK00100/2010/G1P8
N1-RVA/Human-wt/ITA/JES11/2010/G9P8
Human Wa-like stains
N1-RVA/Pig-wt/IND/RU172/2002/G12P7
N1-RVA/Human-tc/BRA/IAL28/1992/G5P8
N1-RVA/Human-wt/JPN/Ryukyu-1120/2011/G5P6
N1-RVA/Pig-wt/JPN/BU2/2014/G5P7
N1-RVA/Pig-tc/KOR/PRG9121/2006/G9P7
N1-RVA/Cat-wt/ITA/BA222/2005/G3P9
N1-RVA/Human-wt/HUN/BP271/2000/G4P6
N1-RVA/Pig-wt/ITA/2CR/2009/G9P23
N2-RVA/Human-tc/USA/DS-1/1976/G2P4
0.05

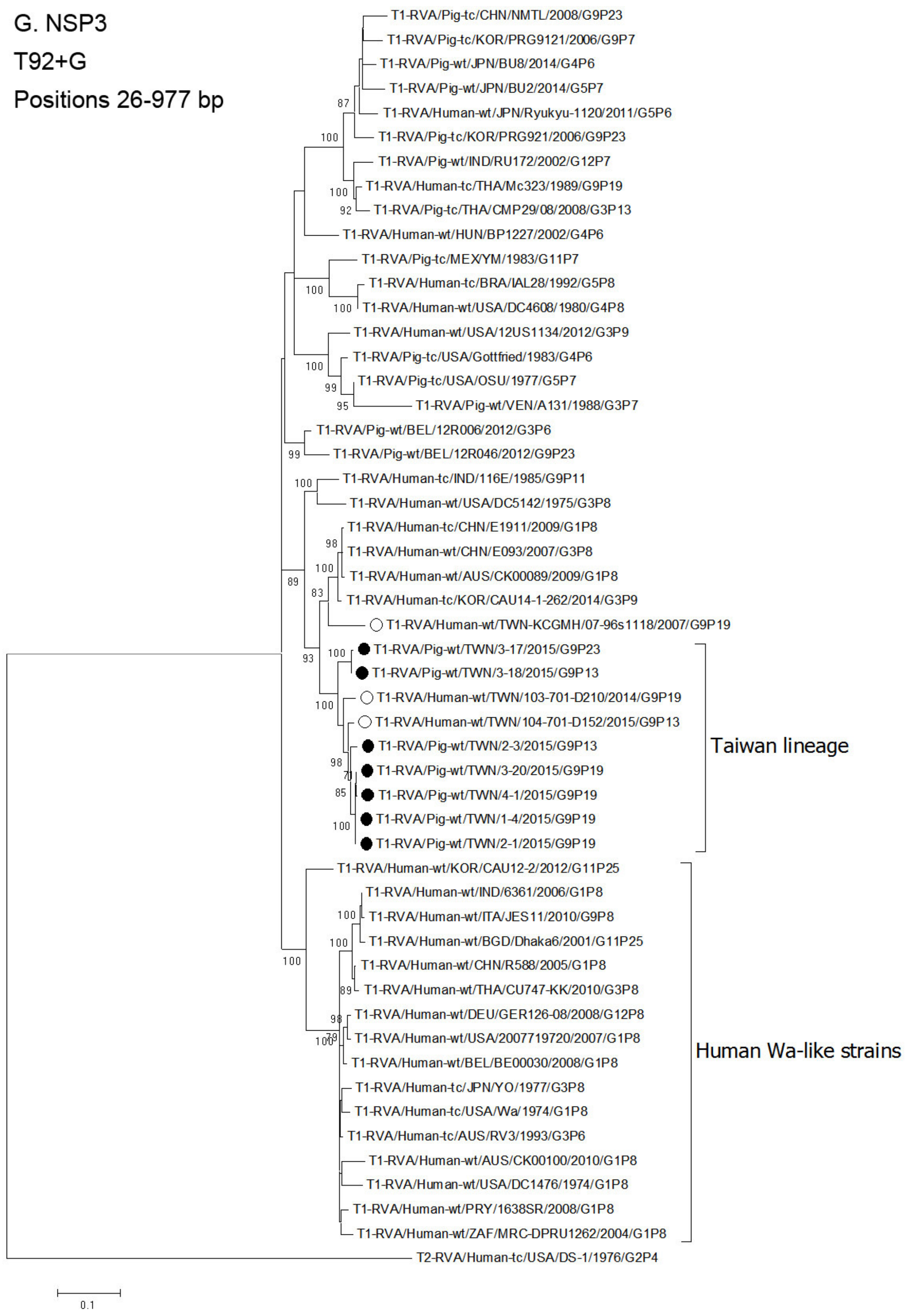
G. NSP3
T92+G
Positions 26-977 bp
T1-RVA/Pig-tc/CHN/NMTL/2008/G9P23
T1-RVA/Pig-tc/KOR/PRG9121/2006/G9P7
T1-RVA/Pig-wt/JPN/BU8/2014/G4P6
T1-RVA/Pig-wt/JPN/BU2/2014/G5P7
T1-RVA/Human-wt/JPN/Ryukyu-1120/2011/G5P6
T1-RVA/Pig-tc/KOR/PRG921/2006/G9P23
T1-RVA/Pig-wt/IND/RU172/2002/G12P7
T1-RVA/Human-tc/THA/Mc323/1989/G9P19
T1-RVA/Pig-tc/THA/CMP29/08/2008/G3P13
T1-RVA/Human-wt/HUN/BP1227/2002/G4P6
T1-RVA/Pig-tc/MEX/YM/1983/G11P7
T1-RVA/Human-tc/BRA/IAL28/1992/G5P8
T1-RVA/Human-wt/USA/DC4608/1980/G4P8
T1-RVA/Human-wt/USA/12US1134/2012/G3P9
T1-RVA/Pig-tc/USA/Gottfried/1983/G4P6
T1-RVA/Pig-tc/USA/OSU/1977/G5P7
T1-RVA/Pig-wt/VEN/A131/1988/G3P7
T1-RVA/Pig-wt/BEL/12R006/2012/G3P6
T1-RVA/Pig-wt/BEL/12R046/2012/G9P23
T1-RVA/Human-tc/IND/116E/1985/G9P11
T1-RVA/Human-wt/USA/DC5142/1975/G3P8
T1-RVA/Human-tc/CHN/E1911/2009/G1P8
T1-RVA/Human-wt/CHN/E093/2007/G3P8
T1-RVA/Human-wt/AUS/CK00089/2009/G1P8
T1-RVA/Human-tc/KOR/CAU14-1-262/2014/G3P9
T1-RVA/Human-wt/TWN-KCGMH/07-96s1118/2007/G9P19
T1-RVA/Pig-wt/TWN/3-17/2015/G9P23
T1-RVA/Pig-wt/TWN/3-18/2015/G9P13
T1-RVA/Human-wt/TWN/103-701-D210/2014/G9P19
T1-RVA/Human-wt/TWN/104-701-D152/2015/G9P13
T1-RVA/Pig-wt/TWN/2-3/2015/G9P13
T1-RVA/Pig-wt/TWN/3-20/2015/G9P19
T1-RVA/Pig-wt/TWN/4-1/2015/G9P19
T1-RVA/Pig-wt/TWN/1-4/2015/G9P19
T1-RVA/Pig-wt/TWN/2-1/2015/G9P19
Taiwan lineage
T1-RVA/Human-wt/KOR/CAU12-2/2012/G11P25
T1-RVA/Human-wt/IND/6361/2006/G1P8
T1-RVA/Human-wt/ITA/JES11/2010/G9P8
T1-RVA/Human-wt/BGD/Dhaka6/2001/G11P25
T1-RVA/Human-wt/CHN/R588/2005/G1P8
T1-RVA/Human-wt/THA/CU747-KK/2010/G3P8
T1-RVA/Human-wt/DEU/GER126-08/2008/G12P8
T1-RVA/Human-wt/USA/2007719720/2007/G1P8
T1-RVA/Human-wt/BEL/BE00030/2008/G1P8
T1-RVA/Human-tc/JPN/YO/1977/G3P8
T1-RVA/Human-tc/USA/Wa/1974/G1P8
T1-RVA/Human-tc/AUS/RV3/1993/G3P6
T1-RVA/Human-wt/AUS/CK00100/2010/G1P8
T1-RVA/Human-wt/USA/DC1476/1974/G1P8
T1-RVA/Human-wt/PRY/1638SR/2008/G1P8
T1-RVA/Human-wt/ZAF/MRC-DPRU1262/2004/G1P8
Human Wa-like strains
T2-RVA/Human-tc/USA/DS-1/1976/G2P4
0.1

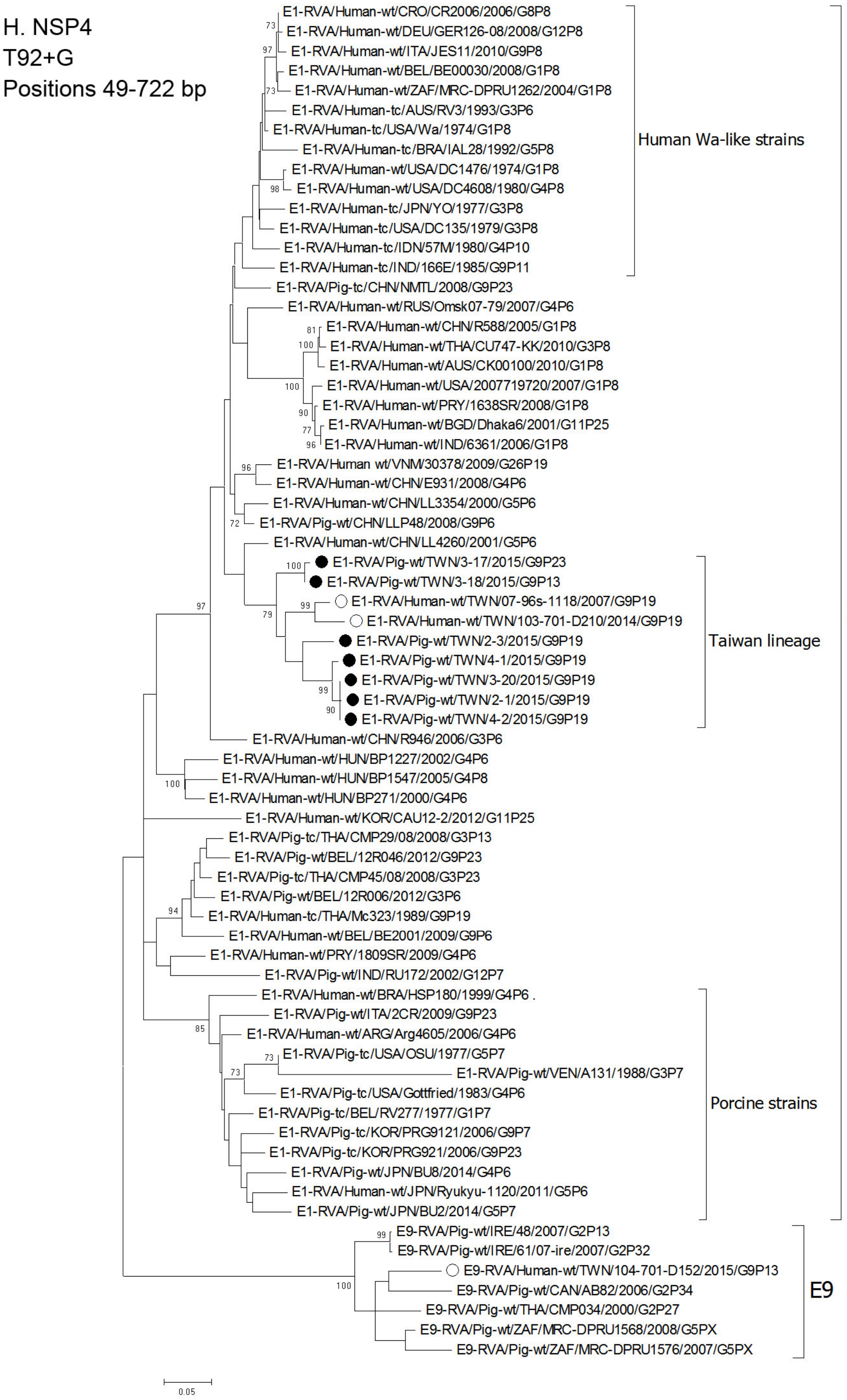

H. NSP4
T92+G
Positions 49-722 bp
E1-RVA/Human-wt/CRO/CR2006/2006/G8P8
E1-RVA/Human-wt/DEU/GER126-08/2008/G12P8
E1-RVA/Human-wt/ITA/JES11/2010/G9P8
E1-RVA/Human-wt/BEL/BE00030/2008/G1P8
E1-RVA/Human-wt/ZAF/MRC-DPRU1262/2004/G1P8
E1-RVA/Human-tc/AUS/RV3/1993/G3P6
E1-RVA/Human-tc/USA/Wa/1974/G1P8
E1-RVA/Human-tc/BRA/IAL28/1992/G5P8
E1-RVA/Human-wt/USA/DC1476/1974/G1P8
E1-RVA/Human-wt/USA/DC4608/1980/G4P8
E1-RVA/Human-tc/JPN/YO/1977/G3P8
E1-RVA/Human-tc/USA/DC135/1979/G3P8
E1-RVA/Human-tc/IDN/57M/1980/G4P10
E1-RVA/Human-tc/IND/166E/1985/G9P11
Human Wa-like strains
E1-RVA/Pig-tc/CHN/NMTL/2008/G9P23
E1-RVA/Human-wt/RUS/Omsk07-79/2007/G4P6
E1-RVA/Human-wt/CHN/R588/2005/G1P8
E1-RVA/Human-wt/THA/CU747-KK/2010/G3P8
E1-RVA/Human-wt/AUS/CK00100/2010/G1P8
E1-RVA/Human-wt/USA/2007719720/2007/G1P8
E1-RVA/Human-wt/PRY/1638SR/2008/G1P8
E1-RVA/Human-wt/BGD/Dhaka6/2001/G11P25
E1-RVA/Human-wt/IND/6361/2006/G1P8
E1-RVA/Human wt/VNM/30378/2009/G26P19
E1-RVA/Human-wt/CHN/E931/2008/G4P6
E1-RVA/Human-wt/CHN/LL3354/2000/G5P6
E1-RVA/Pig-wt/CHN/LLP48/2008/G9P6
E1-RVA/Human-wt/CHN/LL4260/2001/G5P6
E1-RVA/Pig-wt/TWN/3-17/2015/G9P23
E1-RVA/Pig-wt/TWN/3-18/2015/G9P13
E1-RVA/Human-wt/TWN/07-96s-1118/2007/G9P19
E1-RVA/Human-wt/TWN/103-701-D210/2014/G9P19
E1-RVA/Pig-wt/TWN/2-3/2015/G9P19
E1-RVA/Pig-wt/TWN/4-1/2015/G9P19
E1-RVA/Pig-wt/TWN/3-20/2015/G9P19
E1-RVA/Pig-wt/TWN/2-1/2015/G9P19
E1-RVA/Pig-wt/TWN/4-2/2015/G9P19
Taiwan lineage
E1
E1-RVA/Human-wt/CHN/R946/2006/G3P6
E1-RVA/Human-wt/HUN/BP1227/2002/G4P6
E1-RVA/Human-wt/HUN/BP1547/2005/G4P8
E1-RVA/Human-wt/HUN/BP271/2000/G4P6
E1-RVA/Human-wt/KOR/CAU12-2/2012/G11P25
E1-RVA/Pig-tc/THA/CMP29/08/2008/G3P13
E1-RVA/Pig-wt/BEL/12R046/2012/G9P23
E1-RVA/Pig-tc/THA/CMP45/08/2008/G3P23
E1-RVA/Pig-wt/BEL/12R006/2012/G3P6
E1-RVA/Human-tc/THA/Mc323/1989/G9P19
E1-RVA/Human-wt/BEL/BE2001/2009/G9P6
E1-RVA/Human-wt/PRY/1809SR/2009/G4P6
E1-RVA/Pig-wt/IND/RU172/2002/G12P7
E1-RVA/Human-wt/BRA/HSP180/1999/G4P6 .
E1-RVA/Pig-wt/ITA/2CR/2009/G9P23
E1-RVA/Human-wt/ARG/Arg4605/2006/G4P6
E1-RVA/Pig-tc/USA/OSU/1977/G5P7
E1-RVA/Pig-wt/VEN/A131/1988/G3P7
E1-RVA/Pig-tc/USA/Gottfried/1983/G4P6
E1-RVA/Pig-tc/BEL/RV277/1977/G1P7
E1-RVA/Pig-tc/KOR/PRG9121/2006/G9P7
E1-RVA/Pig-tc/KOR/PRG921/2006/G9P23
E1-RVA/Pig-wt/JPN/BU8/2014/G4P6
E1-RVA/Human-wt/JPN/Ryukyu-1120/2011/G5P6
E1-RVA/Pig-wt/JPN/BU2/2014/G5P7
Porcine strains
E9-RVA/Pig-wt/IRE/48/2007/G2P13
E9-RVA/Pig-wt/IRE/61/07-ire/2007/G2P32
E9-RVA/Human-wt/TWN/104-701-D152/2015/G9P13
E9-RVA/Pig-wt/CAN/AB82/2006/G2P34
E9-RVA/Pig-wt/THA/CMP034/2000/G2P27
E9-RVA/Pig-wt/ZAF/MRC-DPRU1568/2008/G5PX
E9-RVA/Pig-wt/ZAF/MRC-DPRU1576/2007/G5PX
E9
0.05

I. NSP5

T92+G

Positions 41-618 bp

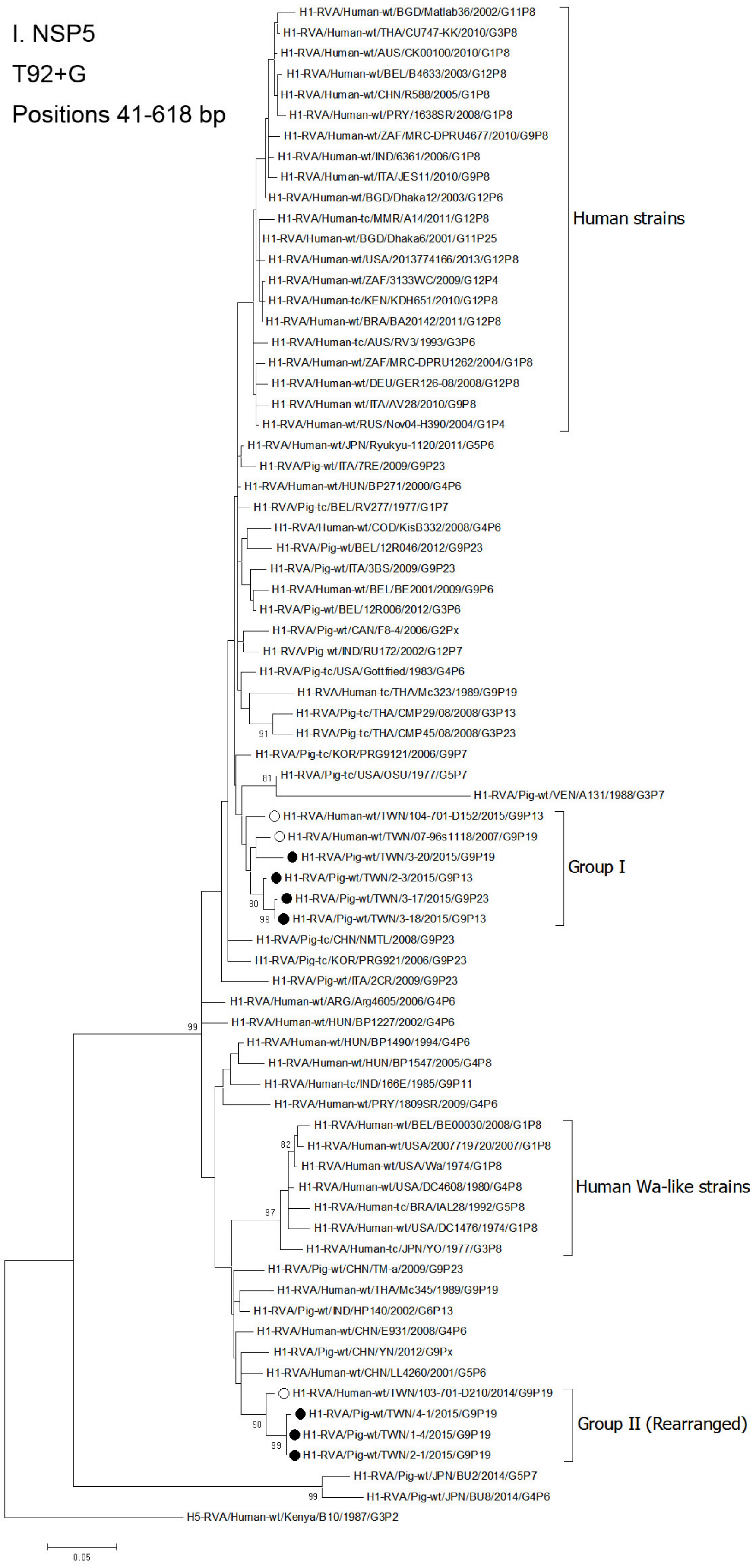

Supplementary Table S1 List of primers used in RT-PCR and sequencing

Target	Name	Sequence	Position (ref accession)	Reference
VP1	**GEN_VP1Fb**	GGC TAT TAA AGC TRT ACA ATG GGG AAG	1-27(DQ490539)	4
	**VP1-771f**	AGT YGC ATT AAT WGA YAT TAA YGG TAC	771-797(DQ490539)	5
	**VP1-955r**	CAA CTA ACC AYT CTT GAA TCA TTC	955-932(DQ490539)	5
	**VP1-1375f**	GAT GTT CCT GGA CGA AGA AC	1375-1394(DQ490539)	5
	**VP1-1619R**	CCC ATW ATT ATT CCT TTY CTR AA	1641-1619(DQ490539)	This study
	**VP1-1650r**	CAT ATC TAA ACC CAT AAT TAT TCC	1650-1627(DQ490539)	5
	**VP1-2216f**	TAA CTA AAA TTA TAC ARA TGA CAT CTG	2216-2242(DQ490539)	5
	**VP1-2294r**	GTW GTT ACW CGT TCT GAY GG	2294-2272(DQ490539)	5
	**GEN_VP1Rb**	GGT CAC ATC TAA GCG YTC TAA TCT TG	3302-3277(DQ490539)	4
VP2	**GEN_VP2Fc**	GGC TAT TAA AGG YTC AAT GGC GTA CAG	1-27 (X14942)	4
	**VP2-m719f**	GAT ATG AGA CAA CAA GTT CAR G	719-740 (X14942)	5
	**VP2-852r**	TTA TTC AAT GGT TCA ACT ART TG	880-858 (X14942)	5
	**VP2-1389f**	GAA TGC AAA GAA TGC ATT ATA G	1368-1389 (X14942)	5
	**VP2-1443r**	AAA TTT TGT ATT TGC TGT TCT GCT A	1443-1419 (X14942)	5
	**VP2-2114f**	GAG CAG ATT GAA CGA GCT TCA	2114-2134 (X14942)	5
	**VP2-2202r**	CCA TAC ATC TCA TCT CTT TC	2202-2183 (X14942)	5
	**GEN_VP2Rbc**	GTC ATA TCT CCA CAR TGG GGT TGG	2716-2693 (X14942)	4
VP3	**GEN_VP3Fe**	GGC TWT TAA AGC ART ATT AGT AGT G	1-25 (AY267335)	4
	**VP3-m758f**	CAT ACT ATY AAA TTG AAK CAR GA	758-780 (AY267335)	5
	**VP3-m913r**	AGC YGA ACC AAG CAT RTA TAY KA	913-891 (AY267335)	5
	**VP3-m1550f**	AAT TTY AAR AAT ATT TAY GAY TGG AC	1550-1575 (AY267335)	5
	**VP3-m1704r**	TTT ATT ATR AAT AAA TGR TTR TTT CC	1704-1679 (AY267335)	5
	**GEN_VP3Rc**	GGT CAC ATC ATG ACT AGT GTG	2591-2570 (AY267335)	4
VP4	**VP4 F primer**	GGC TAT AAA ATG GCT TCG CT	1-20 (JX406750)	6
	**VP4-5a**	AAT GAT TAT CAG ACT CCA AT	1438-1457 (JX406750)	7
	**Con3**	TGG CTT CGC CAT TTT ATA GAC A	11-32 (JX406750)	8
	**VP4F**	TAT GCT CCA GTN AAT TGG	142-159 (JX406750)	15
	**793F**	TGG AAA GAA ATG CAR TAY AA	799-818 (JX406750)	9
	**Con2**	ATT TCG GAC CAT TTA TAA CC	887-868 (JX406750)	8
	**VP4R**	ATT GCA TTT CTT TCC ATA ATG	805-785 (JX406750)	15
	**1583R**	CCH GAR AAC ATN GAR AAC ATA TC	1604-1582 (JX406750)	9
	**VP4 R primer**	GGG GGT CAC ATC CTC	2359-2348 (+3) (JX406750)	6
NSP1	**GEN_NSP1F**	GGC TTT TTT TTA TGA AAA GTC TTG	1-24 (JX406751)	4
	**GEN_NSP1R**	GGT CAC ATT TTA TGC TGC C	1531-1547 (HQ650120)	4
NSP2	**GEN_NSP2F**	GGC TTT TAA AGC GTC TCA G	1-19 (JX406754)	4
	**GEN_NSP2R**	GGT CAC ATA AGC GCT TTC	1059-1042 (JX406754)	4
NSP3	**NSP3 F primer**	GGC TTT TAA TGC TTT TCA GTG GTT G	1-25 (JX406753)	6

	**NSP3 R primer**	GGT CAC ATA ACG CCC CTA TAG	1074-1054 (JX406753)	6
**NSP4**	**GEN_NSP4F**	GGC TTT TAA AAG TTC TGT TCC	1-21 (JX406756)	4
	**JRG30**	GGC TTT TAA AAG TTC TGT	1-18 (JX406756)	10
	**JRG31**	ACC ATT CCT TCC ATT AAG	738-721 (JX406756)	10
	**GEN_NSP4R**	GGW YAC RYT AAG ACC RTT CC	750-731 (JX406756)	4
**NSP5**	**GEN_NSP5F**	GGC TTT TAA AGC GCT ACA G	1-19 (JX406757)	4
	**GEN_NSP5R**	GGT CAC AAA ACG GGA GT	664-648 (JX406757)	4
**VP7**	**Beg9**	GGC TTT AAA AGA GAG AAT TTC CGT CTG G	1-28 (JX406755)	11
	**VP7-39F**	GCT CYT TTT RAT GTA TGG TAT TGA ATA TAC CAC	39-71 (JX406755)	9
	**9con1**	TAG CTC CTT TTA ATG TAT GG	37-56 (JX406755)	12
	**VP7-406R**	CTT TAA AAT ANA DGA DCC WRT YGG CCA	406-379 (JX406755)	9
	**9con2**	GTA TAA AAT ACT TGC CAC CA	941-922 (JX406755)	12
	**End9**	GGT CAC ATC ATA CAA TTC TAA TCT AAG	1062-1036 (JX406755)	11
**VP6**	**GEN_VP6F**	GGC TTT WAA ACG AAG TCT TC	1-20 (JX406752)	4
	**JRG7**	GGC TTT AAA ACG AAG TCT TC	1-20 (JX406752)	14
	**VP6-F**	GAC GGV GCR ACT ACA TGG	747-764 (JX406752)	13
	**VP6-R**	GTC CAA TTC ATN CCT GGT GG	1126-1107 (JX406752)	13
	**JRG8**	GGT CAC ATC CTC TCA CTA CAT	1356-1336 (JX406752)	14
	**GEN_VP6R**	GGT CAC ATC CTC TCA CT	1356-1340 (JX406752)	4

Supplementary Table S2 Percentage identity of each gene segment with reference and closest BLAST hit strains in GenBank

		Human D152 G9P13			Human D210 G9P19			Human 1118 G9P19		
	Cutoff	Genotype	Reference strain %	GenBank strain %	Genotype	Reference strain %	GenBank strain %	Genotype	Reference strain %	GenBank strain %
VP1	83	R1	86% (Wa)	87% (H-1)	R1	86% (Wa)	88% (BU8)	R1	86% (Wa)	88% (BU8)
VP2	84	C1	89% (Wa)	93% (E931)	C1	89% (Wa)	92% (E931)	C1	88% (Wa)	93% (E931)
VP3	81	M1	87% (Wa)	90% (YM1)	M1	86% (Wa)	90% (Mc345)	M1	89% (Wa)	92% (PRG9121)
VP4	80	P13	87% (HP140)	95% (CMP213)	P19	87% (NIV929893)	87% (NIV929893)	P19	88% (NIV929893)	88% (NIV929893)
VP6	85	I12	86% (KTM368)	92% (LL4260)	I12	86% (KTM368)	92% (LL4260)	I12	88% (KTM368)	92% (LL4260)
VP7	80	G9	90% (WI61)	92% (G2275)	G9	91% (WI61)	93% (OM46)	G9	89% (WI61)	92% (OM46)
NSP1	79	A8	83% (Gottfried)	85% (YN)	A8	86% (Gottfried)	92% (TM-a)	A8	86% (Gottfried)	96% (TM-a)
NSP2	85	N1	89% (Wa)	94% (Mc345)	N1	88% (Wa)	95% (Mc345)	N1	89% (Wa)	96% (Mc345)
NSP3	85	T1	90% (Wa)	94% (E931)	T1	90% (Wa)	94% (E931)	T1	90% (Wa)	94% (E931)
NSP4	85	E9	94% (CMP034)	94% (CMP034)	E1	92% (Wa)	94% (Omsk07-79)	E1	93% (Wa)	94% (LL4260)
NSP5	91	H1	94% (Wa)	98% (Ryukyu-1120)	H1	96% (Wa)	97% (TM-a)	H1	94% (Wa)	98% (PRG9121)
		Porcine 2-3 G9P13			Porcine 3-18 G9P13			Porcine 2-1 G9P19		
	Cutoff	Genotype	Reference strain %	GenBank strain %	Genotype	Reference strain %	GenBank strain %	Genotype	Reference strain %	GenBank strain %
VP1	83	R1	87% (Wa)	89% (BP1231)	R1	86% (Wa)	89% (BP1231)	R1	87% (Wa)	92% (CAU12-2)
VP2	84	C1	89% (Wa)	92% (OSU)	C1	89% (Wa)	92% (E931)	C1	88% (Wa)	92% (E931)
VP3	81	M1	87% (Wa)	90% (Mc345)	M1	87% (Wa)	89% (Mc345)	M1	87% (Wa)	91% (Mc345)
VP4	80	P13	87% (HP140)	94% (CMP178)	P13	86% (HP140)	93% (12R041)	P19	87% (NIV929893)	87% (Mc345)
VP6	85	I12	86% (KTM368)	91% (LL4260)	I12	87% (KTM368)	92% (LL4260)	I5	89% (OSU)	93% (BU8)
VP7	80	G9	90% (WI61)	93% (OM46)	G9	91% (WI61)	93% (OM46)	G9	91% (WI61)	92% (OM46)
NSP1	79	A8	84% (Gottfried)	85% (ROTA05)	A8	85% (Gottfried)	91% (TM-a)	A8	84% (Gottfried)	85% (ROTA05)
NSP2	85	N1	89% (Wa)	95% (Mc345)	N1	89% (Wa)	96% (Mc345)	N1	89% (Wa)	94% (Mc345)
NSP3	85	T1	89% (Wa)	94% (GX54)	T1	89% (Wa)	94% (E931)	T1	89% (Wa)	94% (E931)
NSP4	85	E1	93% (Wa)	95% (LL3354)	E1	93% (Wa)	95% (LL4260)	E1	93% (Wa)	94% (E931)
NSP5	91	H1	94% (Wa)	98% (Ryukyu-1120)	H1	93% (Wa)	98% (Ryukyu-1120)	H1	95% (Wa)	97% (LL4260)
		Porcine 4-1 G9P19			Porcine 3-20 G9P19			Porcine 3-17 G9P23		
	Cutoff	Genotype	Reference strain %	GenBank strain %	Genotype	Reference strain %	GenBank strain %	Genotype	Reference strain %	GenBank strain %
VP1	83	R1	87% (Wa)	92% (CAU12-2)	R1	87% (Wa)	89% (DC1476)	R1	86% (Wa)	89% (BP1231)
VP2	84	C1	88% (Wa)	92% (E931)	C1	88% (Wa)	92% (E931)	C1	89% (Wa)	92% (E931)
VP3	81	M1	87% (Wa)	91% (Mc323)	M1	87% (Wa)	91% (Mc323)	M1	86% (Wa)	89% (Mc323)
VP4	80	P19	87% (NIV929893)	87% (Mc345)	P19	87% (NIV929893)	87% (Mc345)	P23	88% (A34)	90% (GUB71)
VP6	85	I5	90% (OSU)	95% (BU8)	I12	86% (KTM368)	91% (LL4260)	I12	87% (KTM368)	92% (LL4260)
VP7	80	G9	91% (WI61)	93% (OM46)	G9	90% (WI61)	92% (OM46)	G9	90% (WI61)	92% (OM46)
NSP1	79	A8	86% (Gottfried)	94% (TM-a)	A8	84% (Gottfried)	89% (TM-a)	A8	90% (Gottfried)	90% (Gottfried)
NSP2	85	N1	89% (Wa)	94% (Mc345)	N1	89% (Wa)	94% (Mc345)	N1	90% (Wa)	96% (Mc345)
NSP3	85	T1	89% (Wa)	93% (GX54)	T1	89% (Wa)	93% (GX54)	T1	90% (Wa)	95% (E931)
NSP4	85	E1	92% (Wa)	94% (LLP48)	E1	92% (Wa)	94% (LL3354)	E1	93% (Wa)	94% (LLP48)
NSP5	91	H1	95% (Wa)	96% (LL4260)	H1	93% (Wa)	98% (Ryukyu-1120)	H1	93% (Wa)	98% (Ryukyu-1120)

Technical Appendix References

1. Wu, F.T., *et al.* Diverse origin of P[19] rotaviruses in children with acute diarrhea in Taiwan: detection of novel lineages of the G3, G5, and G9 VP7 genes. *J. Med. Virol.* **83,** 1279-1287 (2011).
2. Maes, P., Matthijnssens, J., Rahman, M., & Van Ranst, M. RotaC: a web-based tool for the complete genome classification of group A rotaviruses. *BMC Microbiol*. **9,** 238-241 (2009).
3. Tamura, K., Stecher, G., Peterson, D., Filipski, A., & Kumar, S.. MEGA6: Molecular Evolutionary Genetics Analysis version 6.0. *Mol. Biol. Evol*. **30,** 2725-2729 (2013).
4. Matthijnssens, J., *et al.* Full genome-based classification of rotaviruses reveals a common origin between human Wa-Like and porcine rotavirus strains and human DS-1-like and bovine rotavirus trains. *J. Virol.* **82,** 3204-3219 (2008).
5. Okitsu, S., *et al.* Whole-genomic analysis of G3P[23], G9P[23] and G3P[13] rotavirus strains isolated from piglets with diarrhea in Thailand, 2006-2008. *Infect. Genet. Evol.* **18,** 74-86 (2013).
6. Fujii, Y., *et al.* Amplification of all 11 RNA segments of group A rotaviruses based on reverse transcription polymerase chain reaction. *Microbiol. Immunol.* **56,** 630-638 (2012).
7. Cao, X.R., Akihara, S., Fang, Z.Y., Nakagomi, O., & Ushijima, H. Genetic variation in the VP4 and NSP4 genes of human rotavirus serotype 3 (G3 type) isolated in China and Japan. *Microbiol. Immunol.* **43,** 171-175 (1999).

8. Gentsch, J.R., *et al.* Identification of group A rotavirus gene 4 types by polymerase chain reaction. *J. Clin. Microbiol.* **30,** 1365-1373 (1992).
9. Amimo, J.O., Vlasova, A.N., & Saif, L.J.. Detection and genetic diversity of porcine group A rotaviruses in historic (2004) and recent (2011 and 2012) swine fecal samples in Ohio: Predominance of the G9P[13] genotype in nursing piglets. *J. Clin. Microbiol.* **51,** 1142-1151 (2013).
10. Esona, M.D., *et al.* Novel human rotavirus genotype G5P[7] from child with diarrhea, Cameroon. *Emerg. Infect. Dis.***15,** 83-86 (2009).
11. Gouvea, V., *et al.* Polymerase chain reaction amplification and typing of rotavirus nucleic acid from stool specimens. *J. Clin. Microbiol.* **28,** 276-282 (1990).
12. Das, B.K., *et al.* Characterization of rotavirus strains from newborns in New Delhi, India. *J. Clin. Microbiol.* **32,** 1820-1822 (1994).
13. Iturriza-Gomara, M., Wong, C., Blome, S., Desselberger, U., & Gray, J. Rotavirus subgroup characterisation by restriction endonuclease digestion of a cDNA fragment of the VP6 gene. *J. Virol. Methods.* **105,** 99-103 (2002).
14. Le, L.T., *et al.* Development and characterization of candidate rotavirus vaccine strains derived from children with diarhoea in Vietnam. *Vaccine.* **27,** F130-138 (2009).
15. World Health Organization. *Manual of rotavirus detection and characterization methods*. (2009). Availalble at: http://www.who.int/iris/handle/10665/70122.
